# Effect of probiotic intake on athletic ability in healthy people: a systematic review and Bayesian meta-analysis

**DOI:** 10.3389/fnut.2026.1731627

**Published:** 2026-01-30

**Authors:** Xuda Zhang, Zhizhao Chang, Shiao Zhao, Xinfan Wu, Xianfei Wang, Guowen Ai, Ziheng Ning

**Affiliations:** 1 Department of Sports Teaching and Research, Lanzhou University, Lanzhou, China; 2 Faculty of Health Sciences and Sports, Macao Polytechnic University, Macao, Macao SAR, China; 3 School of Physical Education, Hainan Normal University, Haikou, China

**Keywords:** athletic performance, Bayesian meta-analysisathletic performance, endurance, muscle strength, probiotics, VO₂max

## Abstract

**Introduction:**

To address ongoing debate about whether probiotic supplements enhance athletic performance in healthy individuals, and amid uncertainty about effective doses, strain formulations, and population-specific responses, we conducted a prospectively registered systematic review and Bayesian multi-level meta-analysis of randomized controlled trials in healthy adults.

**Methods:**

We searched six databases from inception to 1 July 2025. Two reviewers independently completed study selection, data extraction, and risk of bias assessment using ROB. We synthesized data using hierarchical Bayesian models, and evaluated publication bias with funnel plots, Egger’s test, and significance-based diagnostics.

**Results:**

Twenty-one trials (N = 685) were included. Probiotic supplementation was associated with a small-to-moderate improvement in overall athletic performance [μSMD 0.38, 95% CrI 0.17 to 0.60], with the clearest gains in endurance-centric outcomes, including aerobic endurance [μSMD 0.74, 95% CrI 0.39 to 1.10] and cycle-based VO₂max [μSMD 2.21, 95% CrI 0.64 to 3.68]. Both single-strain [μSMD 0.33, 95% CrI 0.04 to 0.62] and multi-strain [μSMD 0.45, 95% CrI 0.12 to 0.79] regimens were effective. By dose, only the medium tier (1×10⁹ to 1×10¹¹ CFU per day) yielded a significant effect [μSMD 0.38, 95% CrI 0.14 to 0.62]. Significant benefits were observed in athletes [μSMD 0.38, 95% CrI 0.08 to 0.69] and in adults [μSMD 0.46, 95% CrI 0.11 to 0.81]. At the strain level, Lactobacillus plantarum showed a significant effect [μSMD 0.82, 95% CrI 0.12 to 1.50].

**Discussion:**

Probiotic supplementation is associated with a modest yet practically meaningful improvement in athletic performance among healthy adults, with benefits across single- and multi-strain products and the most consistent signal at a medium daily dose. Large multicenter trials with harmonized outcome measures are warranted to refine strain- and dose-specific recommendations. This study was registered with PROSPERO (CRD420251139260).

**Systematic review registration:**

https://www.crd.york.ac.uk/PROSPERO/view/CRD420251139260https://www.crd.york.ac.uk/PROSPERO/view/CRD420251139260, PROSPERO (CRD420251139260).

## Graphical abstract

**Figure:**
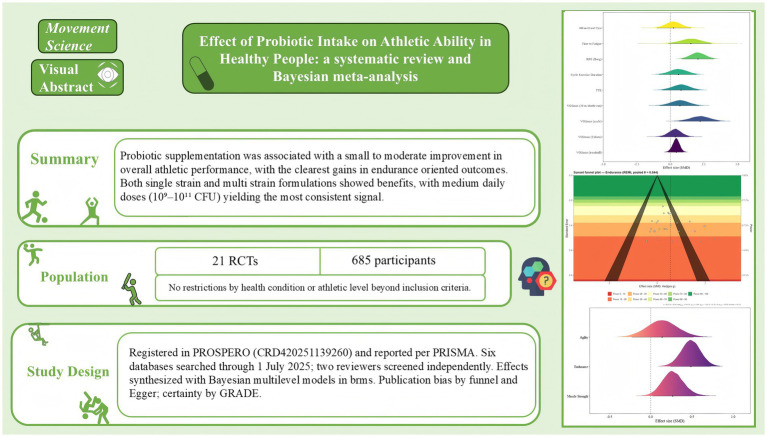

## Introduction

Probiotics are an important component of sports nutrition and are crucial for improving athletic performance in healthy individuals (1). In sports nutrition, the International Society of Sports Nutrition position stand highlights that selected probiotic strains may modulate energy metabolism, enhance nutrient absorption, and regulate oxidative stress and inflammation, providing plausible routes through which performance, particularly strength and endurance, could improve (1–4). However, findings on direct performance gains in healthy individuals remain mixed, suggesting strain and context specific effects and underscoring the need for higher quality studies to determine who benefits, at what dose, and for how long. Accordingly, this study aims to evaluate whether probiotic supplementation affects athletic ability in healthy individuals.

As defined by the FAO/WHO, probiotics are live microorganisms which, when administered in adequate amounts, confer a health benefit on the host (5, 6). Against this backdrop, probiotics as standardizable nutritional interventions with generally good adherence have attracted increasing attention because they may act along the gut-brain-muscle axis to improve substrate availability and mitochondrial function, strengthen mucosal immunity, preserve gut barrier integrity, and modulate neuroendocrine stress responses, thereby reducing fatigue, illness burden, and gastrointestinal symptoms while supporting training capacity and recovery (1, 3, 7). Probiotics show ergogenic potential across both endurance and strength domains. Randomized trials with *Lactiplantibacillus plantarum* TWK10 report longer time to exhaustion (3). Complementary evidence indicates benefits for muscle strength and endurance with *L. plantarum* PL-02, and improved endurance and explosive strength with *Lactococcus lactis* LY-66 (8, 9). Systematic reviews and meta-analyses concur, indicating improvements in aerobic dominant tests and VO₂max, and small but significant gains in global muscle strength, with effects shaped by strain and dose (4, 10). However, the effectiveness of probiotic supplements on athletic performance remains unclear and contentious. Despite a progressively clearer framework, substantial heterogeneity across trials in strains, dose, duration, and population characteristics has yielded inconsistent findings, underscoring the need for rigorous evidence synthesis to clarify both the magnitude of benefit and the conditions under which it arises (1, 11).

Whether probiotic intake improves athletic ability remains debated (11, 12), with several trials and reviews report no improvement in direct performance outcomes such as time to exhaustion, time to fatigue, or marathon finish time, despite some relief of gastrointestinal symptoms (13–16). Null or mixed effects likely reflect transient colonization and strong person specific variability, where many strains fail to engraft in the gut and responses depend on baseline microbiota and diet (17, 18). Benefits are also strain specific and outcome specific, so physiological changes do not always translate into gains in VO₂max, time to exhaustion, or race performance within short study windows (11, 19). Inconsistent dose, duration, and formulation further dilute true effects across trials. Much of the observed benefit appears indirect through immune and gastrointestinal symptom modulation rather than direct enhancement of energy systems or muscle function (12, 20, 21). Additionally, no meta-analysis has systematically evaluated the effects of probiotics in healthy individuals. A rigorous large-scale study is needed to inform supplementation recommendations for this population.

Accordingly, this study aims to evaluate the impact of probiotics on athletic ability in healthy individuals, covering endurance, strength, and speed outcomes, and to identify an appropriate daily dose and formulation. Guided by current but limited evidence, we propose three hypotheses: (1) probiotic supplementation will improve athletic performance, with gains across endurance, muscle strength, and agility; (2) medium daily doses (10^9^ to 10^11^ CFU) and high daily doses (greater than 10^11^ CFU) will yield the most consistent benefits; (3) both single-strain and multi-strain products will be effective, with a favorable signal for *Lactobacillus plantarum*.

## Methods

This study was registered on the PROSPERO platform (registration number: CRD420251139260) and conducted in accordance with the PRISMA guidelines for systematic reviews and meta-analyses (22). During literature screening and data analysis, we used R (version 4.5.1), ASReview LAB v2.1.1 (Python 3), the Covidence platform, and GRADEprofiler to support screening and evaluation procedures (23, 24).

### Literature search

A comprehensive search strategy combining Medical Subject Headings (MeSH) and free-text terms was developed *a priori* and piloted by two authors (XZ and ZC). We systematically searched six databases from inception to July 1, 2025: PubMed, Embase, Web of Science Core Collection, Scopus, EBSCO and Ovid. The final lists of keywords and subject headings were agreed by both reviewers, and the complete search strings for each database are provided in Supplementary File S0. Screening was conducted using the Covidence online platform and ASReview, a Python-based machine learning tool for literature prioritization (23). A total of 4,226 studies were identified for further evaluation.

### Inclusion criteria for screening

Following the PICOS principle, we searched six databases through 1 July 2025. Studies were deemed eligible if they conformed to the PICOS framework criteria. The inclusion criteria were as follows:

Population (P): Healthy humans, including athletes, adults, and older adults.
Intervention (I): Oral probiotic supplementation as the sole active component.
Comparator (C): Placebo with otherwise identical procedures and matched cointerventions.
Outcomes (O): Athletic performance outcomes assessed with validated performance tests.
Study design (S): Randomized controlled trials, either parallel or crossover.

The exclusion criteria were as follows:

Non-original publications (letters, reviews, editorials), theses and conference abstracts; studies without extractable performance data; trials with unmatched cointerventions; multi-ingredient products in which the independent probiotic effect could not be isolated; non-oral delivery; clinical or disease-based populations; and articles not published in English.

### Screening process

All titles and abstracts were first evaluated with ASReview, a machine learning based screening tool. ASReview predicts study relevance by training a classification model on labeled abstracts and continuously reprioritizes the remaining records according to their likelihood of inclusion (23, 25, 26). This approach markedly reduces manual workload by presenting the most likely relevant records first.

During this phase we applied the conservative SAFE rule, which stops screening only after 200 consecutive records have been judged irrelevant (24). Full-text screening was then conducted independently by two authors (XZ and ZC) on the Covidence platform, as recommended in PRISMA guidelines. Eligible articles were recorded with the Extraction 1.0 form, and any disagreements were resolved by a third and fourth author (SZ and ZN) (27). To ensure reproducibility, the ASReview project export is provided in Supplementary File S1.

### Extract data information

For each study, the characteristics extracted included authors, publication year, country, intervention, probiotic supplementation, dose and duration, study design, sample size, sex, athlete status, age, training years, and outcomes. Athletic-performance outcomes included peak torque (PT) at 60°/s and 180°/s in knee extensors (KE) and knee flexors (KF); average peak torque (AvPT) at 60°/s and 180°/s in KE and KF; time to peak torque (TPT) at 60°/s and 180°/s in KE and KF; average rate of force development (AvRFD) at 60°/s and 180°/s in KE and KF; 1RM squat; vertical jump height (cm); PT 60°/s in the knee; relative peak torque; RFD 60°/s in the knee; 30 s chair-stand test (30CST); countermovement jump (CMJ); knee flexor strength; knee extensor strength; 1RM deadlift; vertical jump power (W); standing long jump (SLJ); Running-based Anaerobic Sprint Test (RAST); peak and mean power output in cycling (PPO, MPO); 30 s Wingate test (WAnT 30 s); continuous reverse vertical jump (endurance); individual anaerobic threshold (IAT); Ronaldo Speed Test; handgrip strength left, right, and total (HGS left, HGS right, HGS); hand muscle strength; 1RM bench press; 60 s pull-ups (repetitions); 10 m walk test (10MWT); 40 m dash; 100 m sprint (round trip); sprint test; 10 yard sprint; VO₂max (treadmill, Tabata, cycle, 20 m shuttle run); time to exhaustion (TTE); cycle exercise duration; rating of perceived exertion (RPE, Borg); time to fatigue; and 400 m crawl test.

Data were separately extracted by two authors (XZ and ZC) with Covidence, with discrepancies addressed by consultation with the third and fourth authors (SZ and ZN). Data were expressed as mean ± standard deviation (M ± SD). We utilized meta-analysis accelerator to transform data that were not originally in mean ± SD format (28). When data were not presented as exact numbers, GetData Graph Digitizer was used to extract values from figures (29). Since none of the studies provided correlation coefficients, a correlation of 0.5 was assumed for all trials, following the recommendation of Follmann et al. (30).

The Metafor package in R was used to calculate the standardized mean difference (SMD) according to the formula:


$$\mathrm{SMD}=\sqrt{\frac{(n1i-1)*\mathrm{sd}1i^{2}+(n2i-1)*\mathrm{sd}2i^{2}}{n1i+n2i-2}}$$

### Risk bias assessment

The risk of bias in all included studies was independently evaluated according to the criteria in the Cochrane Handbook of Systematic Reviews of Interventions (31). Two authors (XZ and ZC) evaluated the studies in randomized controlled trials (RCTs) through the Covidence tool in accordance with the Cochrane Risk of bias Assessment Criteria (ROB), covering seven areas of bias: (1) Random sequence generation; (2) Allocation concealment (3) Blinding of participants and staff; (4) Blinding of outcome assessment; (5) Incompleted data; (6) Selective reporting (7) Other biases. The risk of bias is classified as low, unclear or high. All the assessment results were agreed upon through discussion and recorded in the Excel template. Subsequently, the data were input into the R software, and the bias risk summary graph was generated using the robvis package (32). Studies with more than two but fewer than four categories classified as unclear risk were deemed to have moderate overall risk.

Furthermore, funnel plots were produced utilizing the PublicationBias package (33), to detect any publication bias existent in the included studies. The graphs depict both affirmative and non-affirmative studies as round dots located on each side of the funnel’s central axis, with a black diamond representing the overall effect size. The grey diamond signifies the comprehensive estimate derived from the non-affirmative study. By tuning the favor_positive option to TRUE, the study focuses directly on the bias towards positive outcomes, providing a more coherent appreciation of any potential bias in the literature favoring significant discoveries. Evaluating the symmetry of the data points aids in identifying the existence of publication bias. The Egger test was conducted using the rma.mv function from the metafor package. A *p*-value below 0.05 signifies the existence of possible risk bias. The S value, obtained from the PublicationBias package, signifies the magnitude of publication bias necessary to nullify the meta-analytic effect (33).

### Statistical analysis

Bayesian mixed-effects models, executed via the brms package (34) in R 4.5.1, were employed to analyze variation in effect sizes. Given the inclusion of multiple outcomes per study, a two-level model was implemented: level 1 (effect sizes within studies), level 2 (study-level variation). Random intercepts were specified at both levels to account for clustering and study-specific variability. We constructed models assuming a normal distribution and included random effects for within-study ID and between-study ID to account for heterogeneity in each outcome, utilizing two formulas:


$$\begin{array}{l} I_{\text{within}}^{2}=\frac{\tau_{\text{withID}}^{2}}{\text{total}\_\text{variance}}\times 100\\ I_{\text{between}}^{2}=\frac{\tau_{\text{betweenID}}^{2}}{\text{total}\_\text{variance}}\times 100 \end{array}$$

In addition to heterogeneity indicators, we tested two indices through the metainc package, including the dissimilarity index (DI) and the across-studies inconsistency (ASI) to assess the potential inconsistency in each model. These indices were considered more suitable for Bayesian meta-analysis compared to traditional heterogeneity indicators such as *I*^2^ or *Q*-test (35). A DI ≥50% and an ASI ≥25% were considered indicative of important inconsistency. These indices differ from previously existing measures by considering effect size (ES) in the context of decision thresholds (DTs). We set three decision thresholds as suggested for SMD, namely 0.2, 0.5, and 0.8 (35).

Weakly informative priors (mean = 2, sd = 0.5) were used for the random effects. While not universally optimal, weakly informative priors are widely regarded as a best-practice default in Bayesian meta-analysis, especially when prior knowledge is limited (36). Based on our grouping scheme, we fitted 15 Bayesian models using brms to interrogate athletic performance outcomes and reduce residual heterogeneity. Model definitions were refined iteratively in response to reviewer feedback and diagnostic checks, ensuring specification errors were corrected and fit was improved. The models were as follows:

1. Null model to estimate overall effect sizes. This model is structured around athletic ability outcomes and assesses the effects and heterogeneity of probiotic supplementation.
2. Demographic model. This model categorizes participants as Athlete, Adults, and Older adults to examine effects and heterogeneity of probiotic supplementation.
3. Probiotic formulation type model. This model categorizes interventions by probiotic formulation type [single-strain probiotics (SSP) vs. multi-strain probiotics (MSP)] to examine effects and heterogeneity of probiotic supplementation.
4. Single-strain model. This model categorizes interventions that use a single probiotic strain: *Bacillus subtilis*, *Bifidobacterium animalis*, *Lacticaseibacillus paracasei*, *Lactiplantibacillus brevis*, *Lactobacillus casei*, *Lactobacillus helveticus*, *Lactobacillus plantarum*, *Saccharomyces boulardii*.
5. Probiotic dosage model. This model categorizes interventions by daily dose: low (<1 × 10^9^ CFU/day), medium (1 × 10^9^–1 × 10^11^ CFU/day), high (>1 × 10^11^ CFU/day) (1).
6. Muscle strength model. This model further categorizes muscle strength outcomes to explore the sources of effect and heterogeneity of probiotic supplementation.
7. Agility model. This model further categorizes agility outcomes to explore the sources of effect and heterogeneity of probiotic supplementation.
8. Endurance model. This model further categorizes endurance outcomes to explore the sources of effect and heterogeneity of probiotic supplementation.
9. Lower limb strength model. This model further categorizes lower limb strength to explore the sources of effect and heterogeneity of probiotic supplementation.
10. Upper limb strength model. This model further categorizes upper limb strength outcomes to explore the sources of effect and heterogeneity of probiotic supplementation.
11. Anaerobic endurance model. This model further categorizes anaerobic-endurance outcomes to explore the sources of effect and heterogeneity of probiotic supplementation.
12. Change of direction model. This model further categorizes change of direction outcomes to explore the sources of effect and heterogeneity of probiotic supplementation.
13. Sprint speed model. This model further categorizes sprint speed outcomes to explore the sources of effect and heterogeneity of probiotic supplementation.
14. Aerobic endurance model. This model further categorizes aerobic endurance outcomes to explore the sources of effect and heterogeneity of probiotic supplementation.
15. Financial support model. This model categorizes studies by funding status (unfunded vs. funded) to examine effects and heterogeneity of probiotic supplementation.

Four MCMC chains with 10,000 iterations each were run per model. Convergence was evaluated solely with the Rhat statistic, which contrasts between-chain and within-chain variance; values close to 1 indicate adequate convergence. Evidence for a nonzero effect was quantified with Bayes factors (BFs). We computed BFs using bayesfactor_parameters in the bayestestR package, which implements the reciprocal of the Savage Dickey density ratio. Interpretation followed conventional thresholds: BF >3 denotes moderate evidence and BF >10 indicates strong evidence for an effect (37). For hierarchical models, estimating the 95% HDI is seamless because it comes straight from the posterior; by contrast, *p* values and confidence intervals depend on further assumptions and approximations (38).

### Moderation analysis

Analyses were conducted using the brms package (Bayesian framework), with four prespecified moderators: gender, age, weight, and sample size. We also included session duration as a dose related moderator to assess how intervention length influences athletic performance under probiotic supplementation. We first fit a null model (no moderators), then fit single-moderator models (one model per moderator). Posterior summaries are reported as μ (SMD) with 95% credible intervals (CrI); we interpreted standardized mean differences (SMDs) using conventional benchmarks (≈0.2 small, ≈0.5 moderate, ≈0.8 large) (39); The 95% credible interval (CrI) represents the range within which the true effect lies with 95% posterior probability given the model and priors (40); priors and the hierarchical structure matched the main analysis To compare explanatory power, we computed Bayesian *R*^2^ for each model using Bayes *R*^2^ and visualized the posterior distributions of *R*^2^ with density plots, enabling direct comparison of each moderator model against the null model. This Bayesian approach estimates effect sizes while accounting for measurement error and the multilevel data structure.

### Certainty in evidence

The quality of the probiotic evidence was assessed using the GRADE approach, which evaluates the risk of bias, inconsistency, indirectness, and imprecision of effect estimates. The GRADE approach classifies the quality of evidence as high, moderate, low, or very low (31). Furthermore, in the evaluation of inconsistency, in addition to the heterogeneity indicator *τ* (tau), we also used the previously calculated inconsistency indicator (DI & ASI) to help judge the quality of the results. If the inconsistency indicator showed moderate or above inconsistency, the level of the results was downgraded by at least one level.

## Results

The results are presented in seven sections as follows: study selection, characteristics of included studies, quality assessment, meta-analysis using 15 models, moderation analysis, quality grading for each outcome, and publication bias.

### Study selection

**Figure 1:**
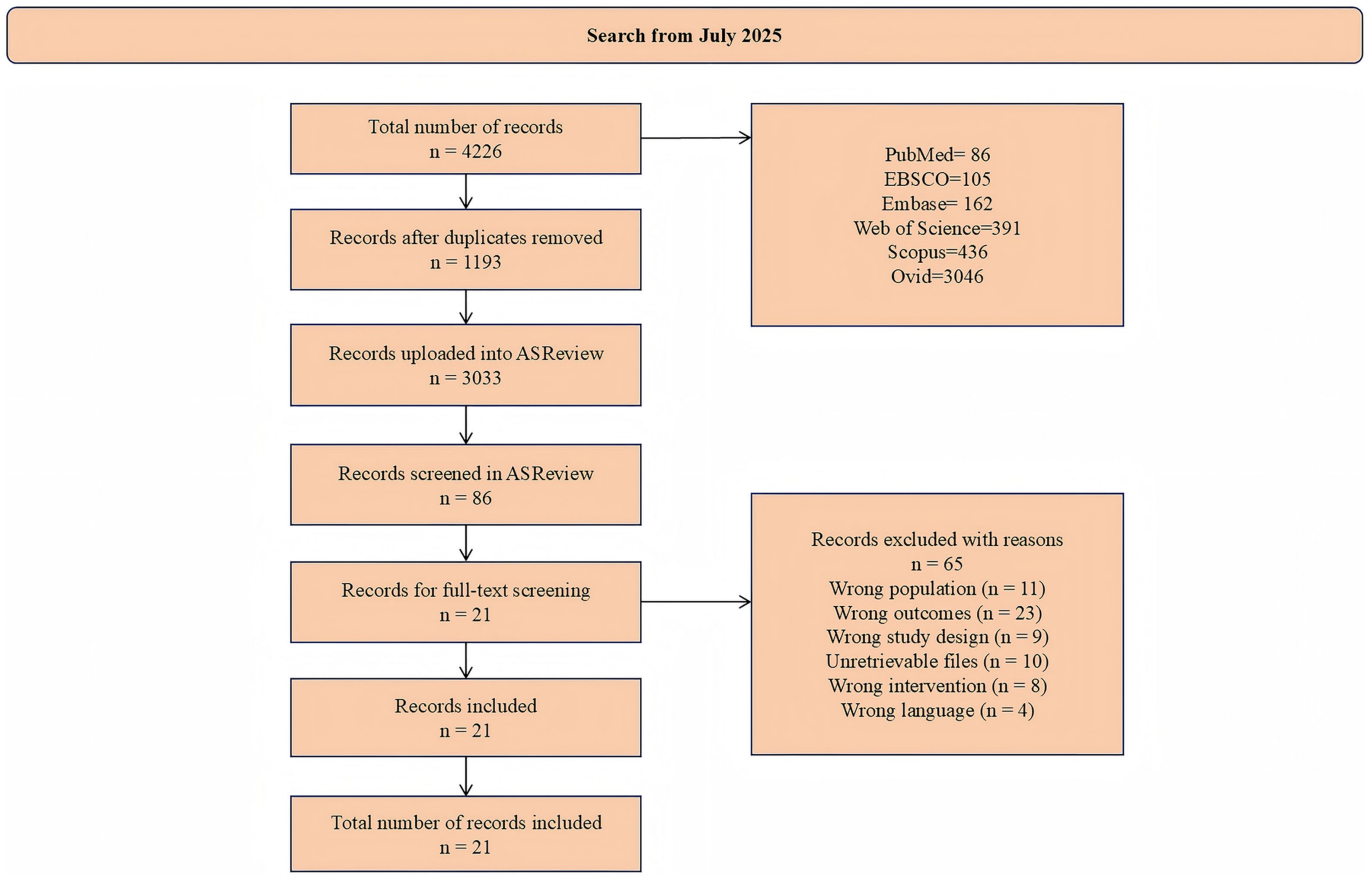
PRISMA flow chart for the identification of the included studies.

Figure 1 illustrates the flow chart. A total of 4,226 articles were extracted from six databases and imported into EndNote for de-duplication. After de-duplication, 3,033 records were imported into ASReview for preliminary title and abstract screening. Using machine-learning prioritization, 504 records were manually labeled at the title and abstract stage, and 86 records were labeled as potentially relevant and retrieved for full-text assessment. Following full-text screening, 21 studies met the eligibility criteria and were incorporated into the meta-analysis. Records excluded with reasons are detailed in Figure 1 and Supplementary File S1.

### Characteristics of included studies

The specific characteristics of each study are provided in Supplementary File S2. A total of 21 studies were included. By continent, Asia accounted for 63.6%, Europe 13.6%, America 18.2%, and Oceania 4.5%. Regarding probiotic formulations at the study level, single-strain (SSP) trials comprised 12 studies (3, 14, 19, 21, 41–47), and multi-strain (MSP) trials comprised 9 studies (8, 46, 48–54). With respect to sex composition, male only studies numbered 9, female only 2 (21, 54), mixed-sex 9, and one study did not report sex information. For population characteristics, athlete samples were used in 12 studies, adult (non-athlete) samples were used in 8 studies, and older-adult samples were used in 1 study (42). Concerning funding, 13 studies reported funding support, and 8 reported no funding. Regarding intervention forms, 16 studies used capsule, 3 used drink (8, 21, 45), 2 used yogurt (44, 54).

### Quality assessment

**Figure 2:**
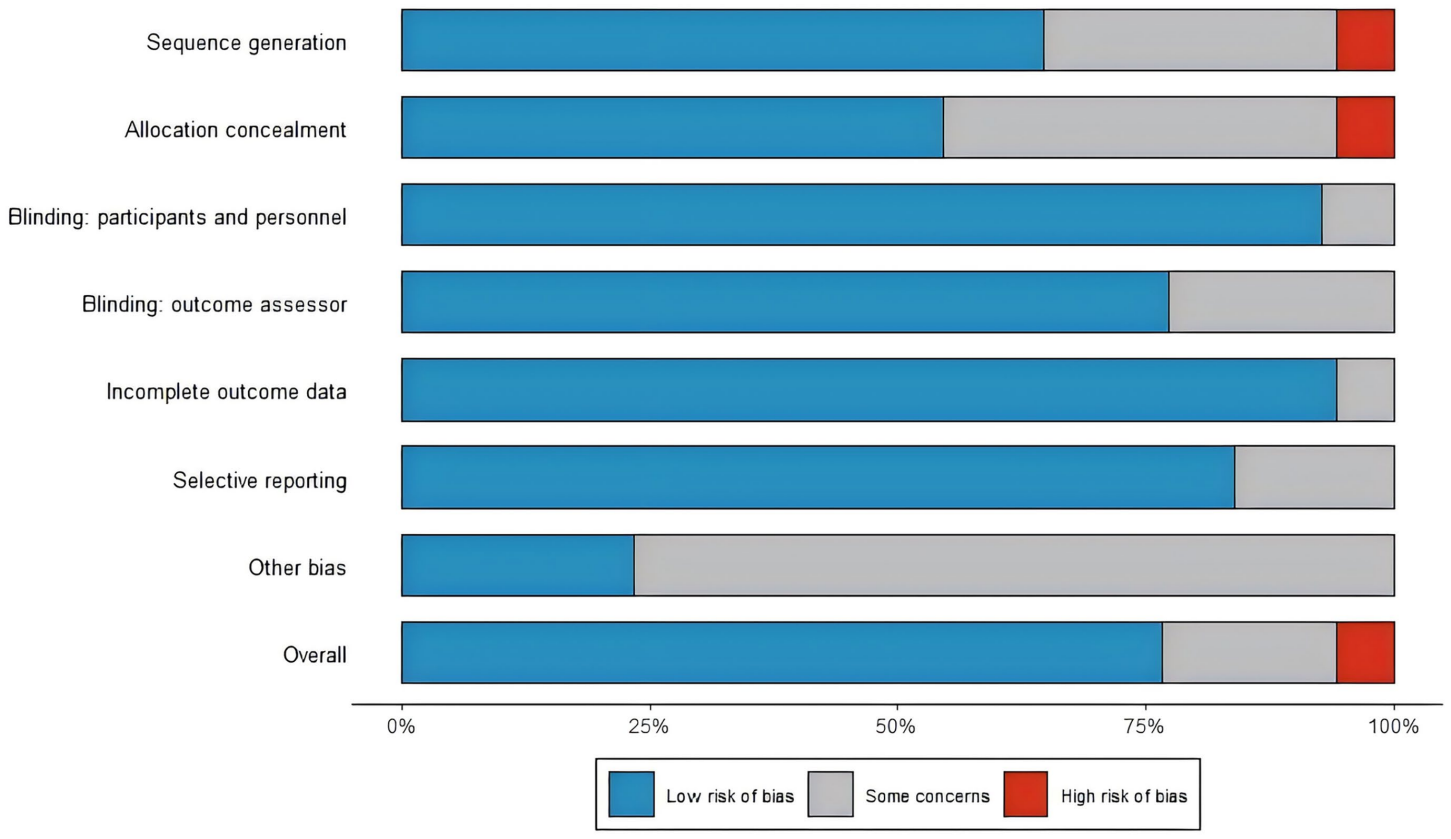
Risk of bias summary.

The risk-of-bias summary is illustrated in Figure 2, with the study-level graph in Supplementary File S3. A small proportion were judged high risk in randomization domains sequence generation: 9.1% and allocation concealment: 9.1% due to problems such as inappropriate block randomization. Blinding (participants) was low risk in 90.9% of studies (remaining 9.1% some concerns; 0% high risk), and blinding of outcome assessors was low risk in 77.3% (22.7% some concerns; 0% high risk). Incomplete outcome data showed 95.5% low risk [4.5% some concerns (48); 0% high risk], and selective reporting was 86.4% low risk (13.6% some concerns, 0% high risk). Other bias was primarily some concerns (72.7%) with 27.3% low risk (0% high risk). Overall, judgments were 81.8% low risk, 13.6% some concerns (3, 19, 44, 45, 47, 54), and 4.5% high risk (48).

### The results of meta-analysis

The meta-analysis is divided into 15 sections. The detailed results for each model are illustrated in Supplementary File S4. The convergence of the Markov chains across all 15 models was evaluated using the Rhat statistic; the Rhat values for all outcomes were approximately 1.0. Therefore, we did not include additional findings on Markov chain convergence in the article.

### Null model

**Figure 3:**
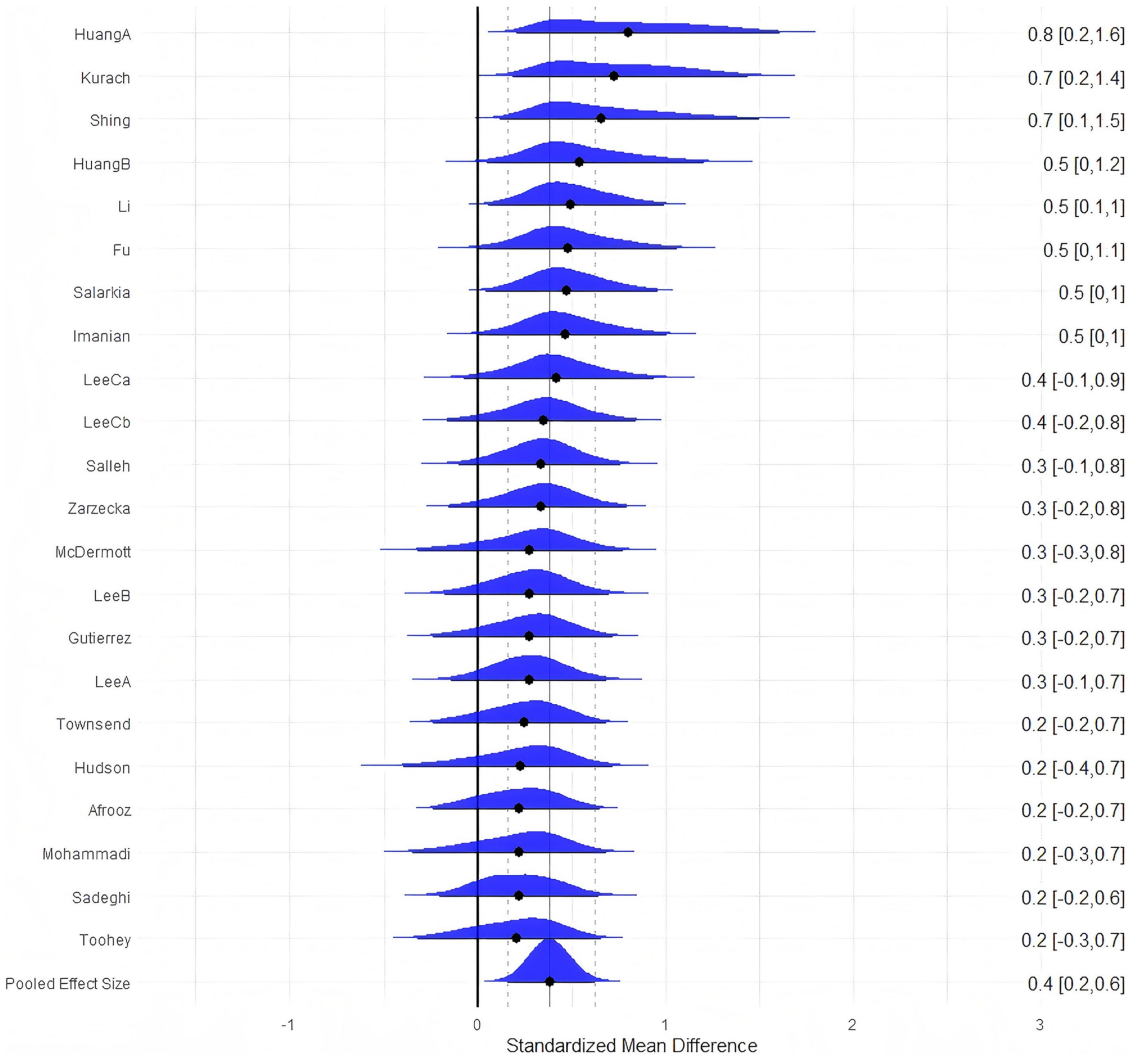
The forest plot in null model.

In the null model, the overall effect size was calculated for athletic ability. A total of 685 participants were included, and the forest plot is illustrated in Figure 3. The Bayesian meta-analysis showed a statistically significant effect [μ(SMD) = 0.38, 95% CrI: 0.17–0.60; 95% HDI: 0.17–0.60; BF_10_ = 12.23], with heterogeneity parameters *τ*_within_ = 0.27 and *τ*_between_ = 0.27.

**Figure 4:**
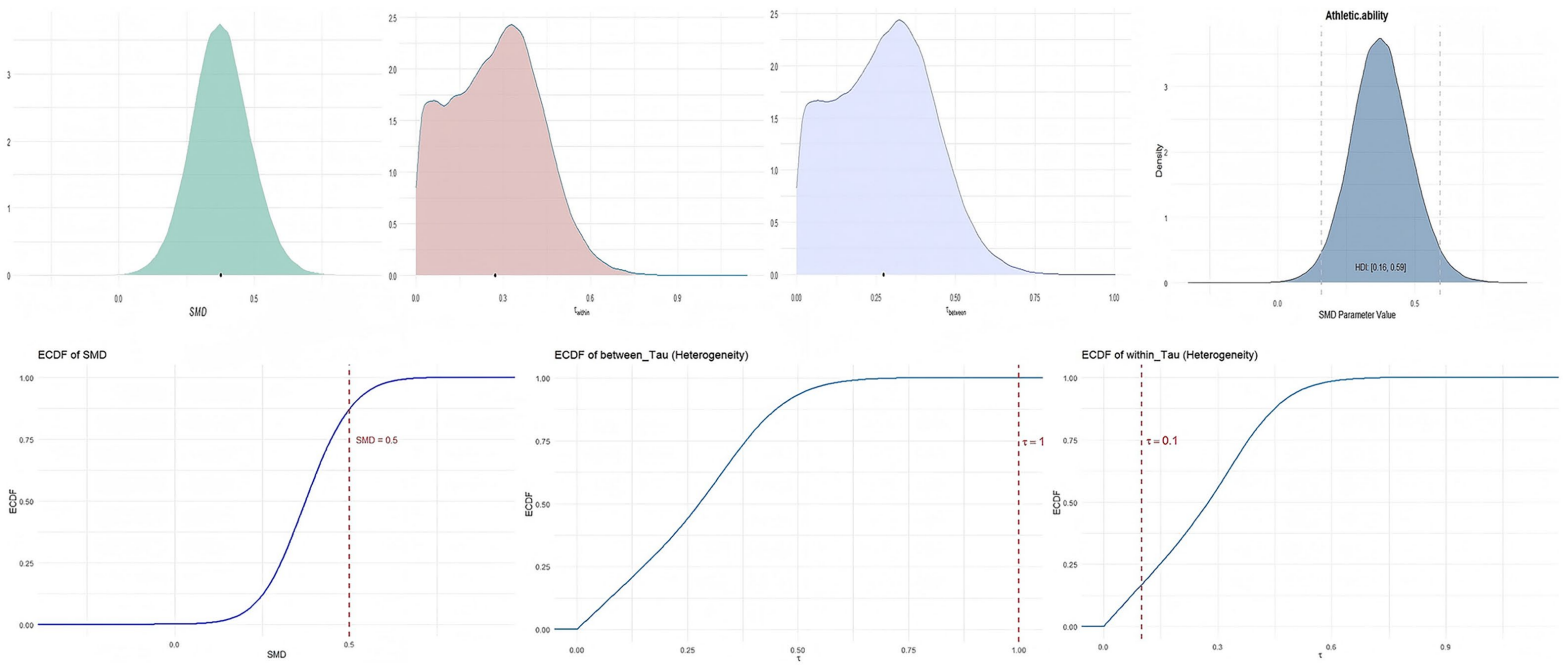
The cumulative probability, HDI distribution, and posterior density distribution (SMD, *τ*_within,_ and *τ*_between_) in athletic ability.

Probiotic supplementation can effectively improve athletic performance in healthy individuals. Figure 4 shows the cumulative probability and posterior density distributions of SMD, *τ*_within_, and *τ*_between_ in athletic performance. As illustrated in Figure 4, the posterior density distributions of *τ*_between and *τ*_within indicate low-to-moderate heterogeneity (*τ* ≈ 0.27). Additionally, the posterior distribution of the standardized mean difference (SMD) suggests a small-to-moderate effect (SMD ≈ 0.38). An SMD of 0.38 indicates that, on average, the probiotic group performed 0.38 standard deviations higher than control across heterogeneous performance tests. Under conventional benchmarks, the posterior mean falls between a small (0.2) and moderate (0.5) effect, while the 95% CrI (0.17–0.60) spans from very small to moderate improvements.

### Demographic model

**Figure 5:**
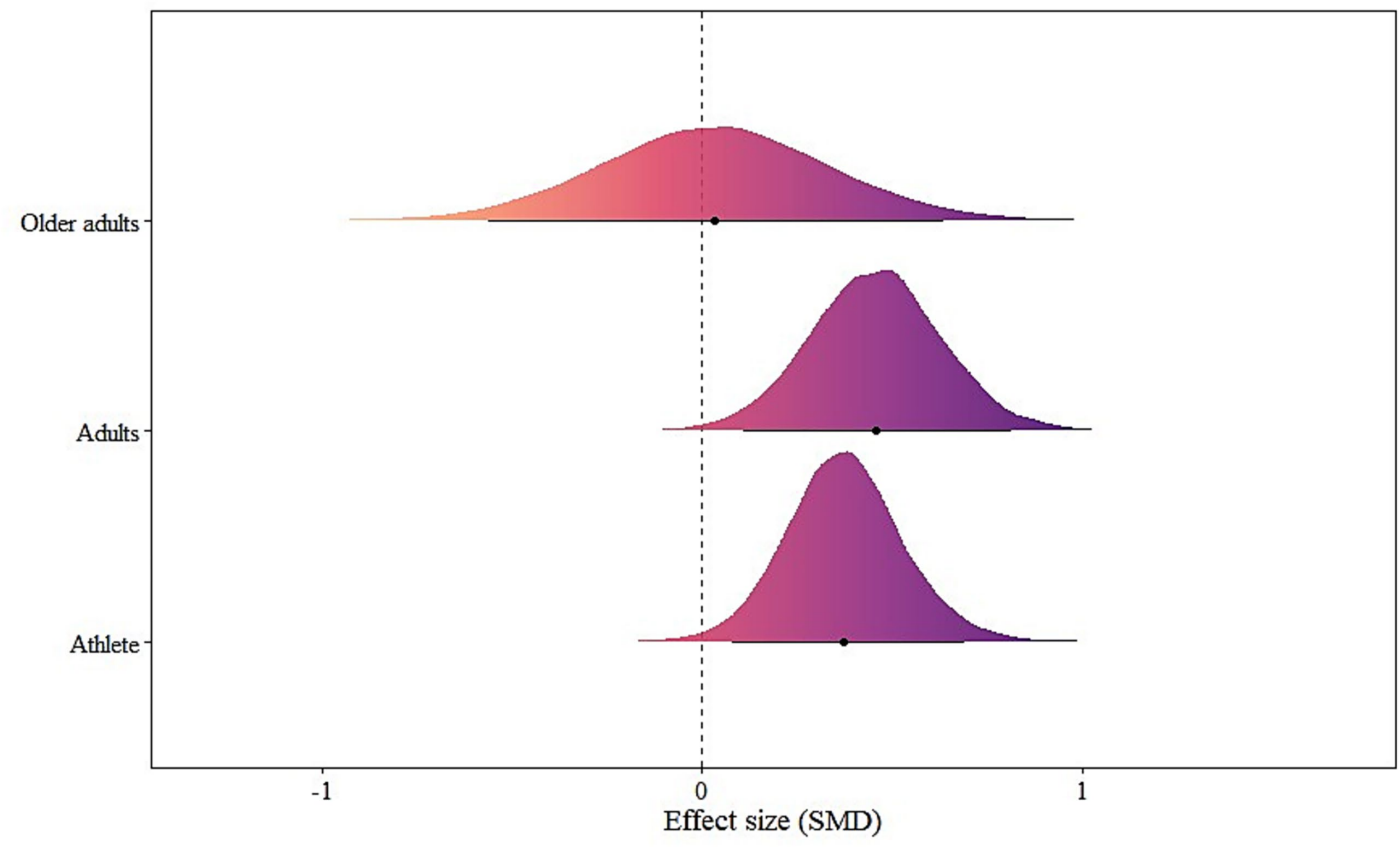
The forest plot in demographic model.

Significant effects were found for Athlete [μ(SMD): 0.38, 95% CrI: 0.08 to 0.69; HDI: 0.08 to 0.69; BF: 1.66] and Adults [μ(SMD): 0.46, 95% CrI: 0.11 to 0.81; HDI: 0.11 to 0.81; BF: 2.36]. Older adults were not statistically significant. Heterogeneity was small (*τ*_within_: 0.29; *τ*_between_: 0.29) (see Figure 5).

### Probiotic formulation type model

**Figure 6:**
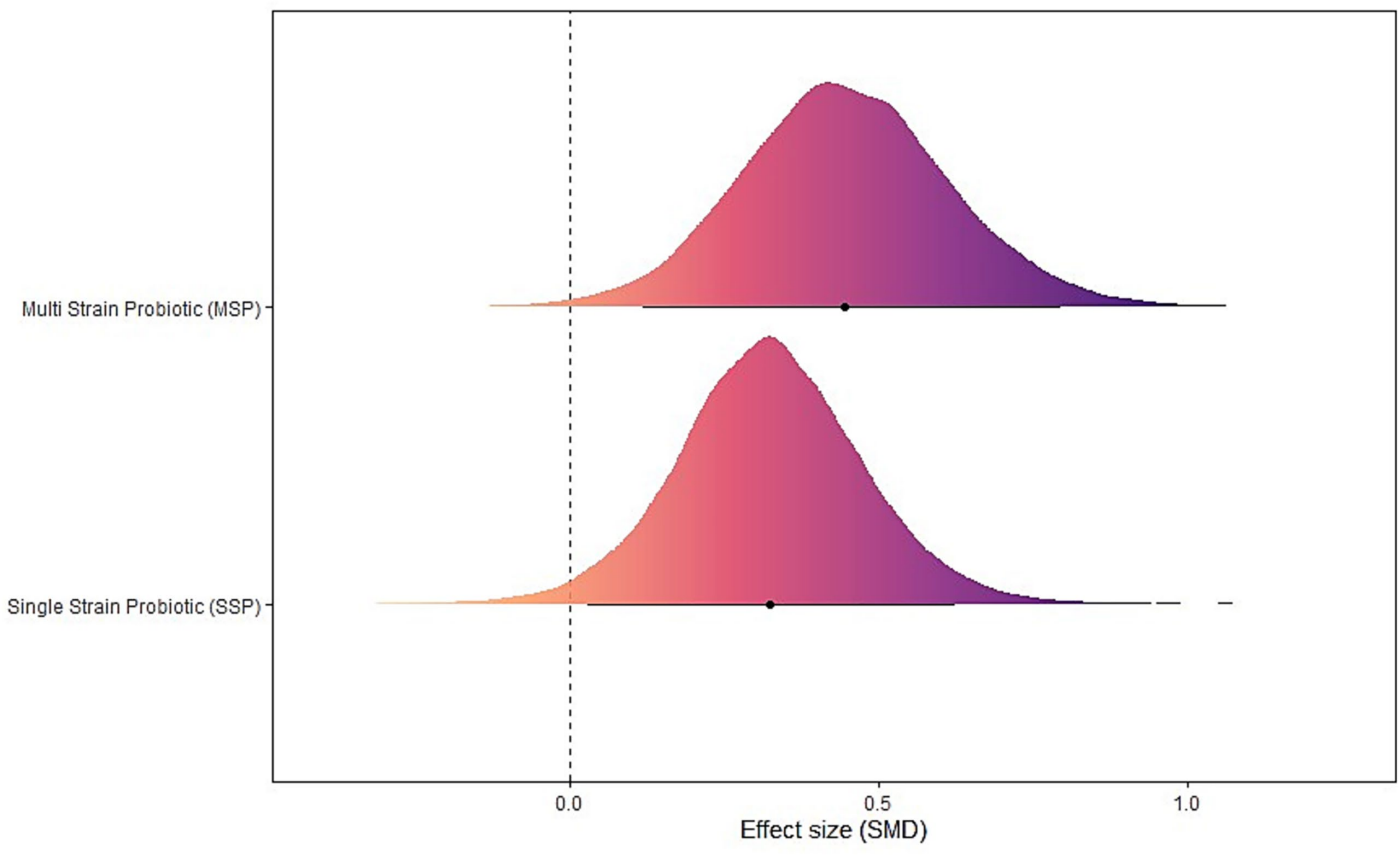
The forest plot in probiotic formulation type model.

Both formulations showed significant effects: single-strain probiotics (SSP) [μ(SMD): 0.33, 95% CrI: 0.04 to 0.62; HDI: 0.03 to 0.62; BF: 0.91] and Multi-strain probiotics (MSP) [μ(SMD): 0.45, 95% CrI: 0.12 to 0.79; HDI: 0.12 to 0.79; BF: 2.65]. Heterogeneity was small (*τ*_within_: 0.28; *τ*_between_: 0.28) (see Figure 6).

### Single-strain probiotics model

**Figure 7:**
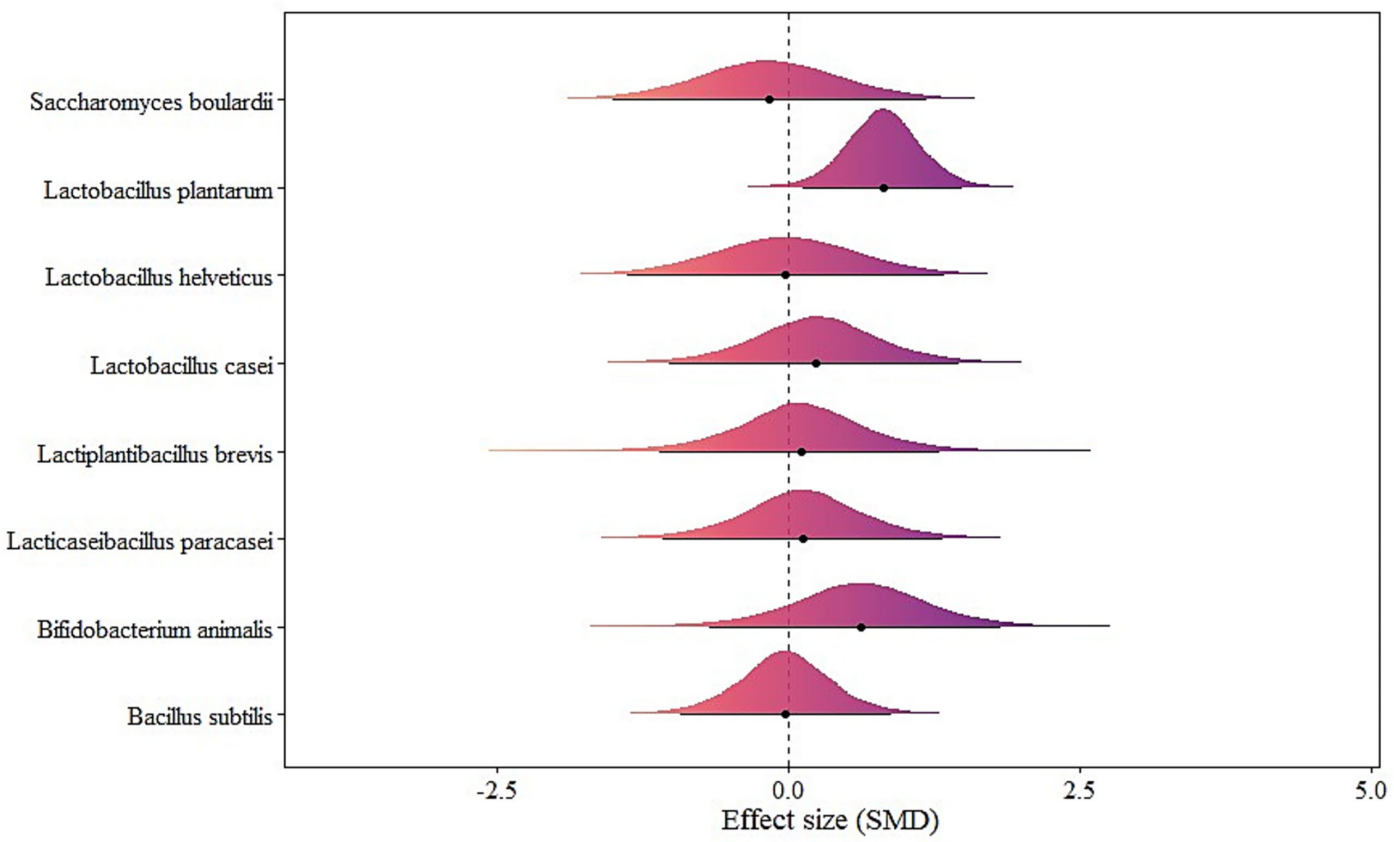
The forest plot in single-strain probiotics model.

Only *Lactobacillus plantarum* showed a statistically significant effect [μ(SMD): 0.82, 95% CrI: 0.12 to 1.50; HDI: 0.14 to 1.52; BF: 2.83]. All other single-strains (*Bacillus subtilis*, *Bifidobacterium animalis*, *Lacticaseibacillus paracasei*, *Lactiplantibacillus brevis*, *Lactobacillus casei*, *Lactobacillus helveticus*, *Saccharomyces boulardii*) were not statistically significant. Heterogeneity was small (*τ*_within_: 0.36; *τ*_between_: 0.36) (see Figure 7).

### Probiotic dosage model

**Figure 8:**
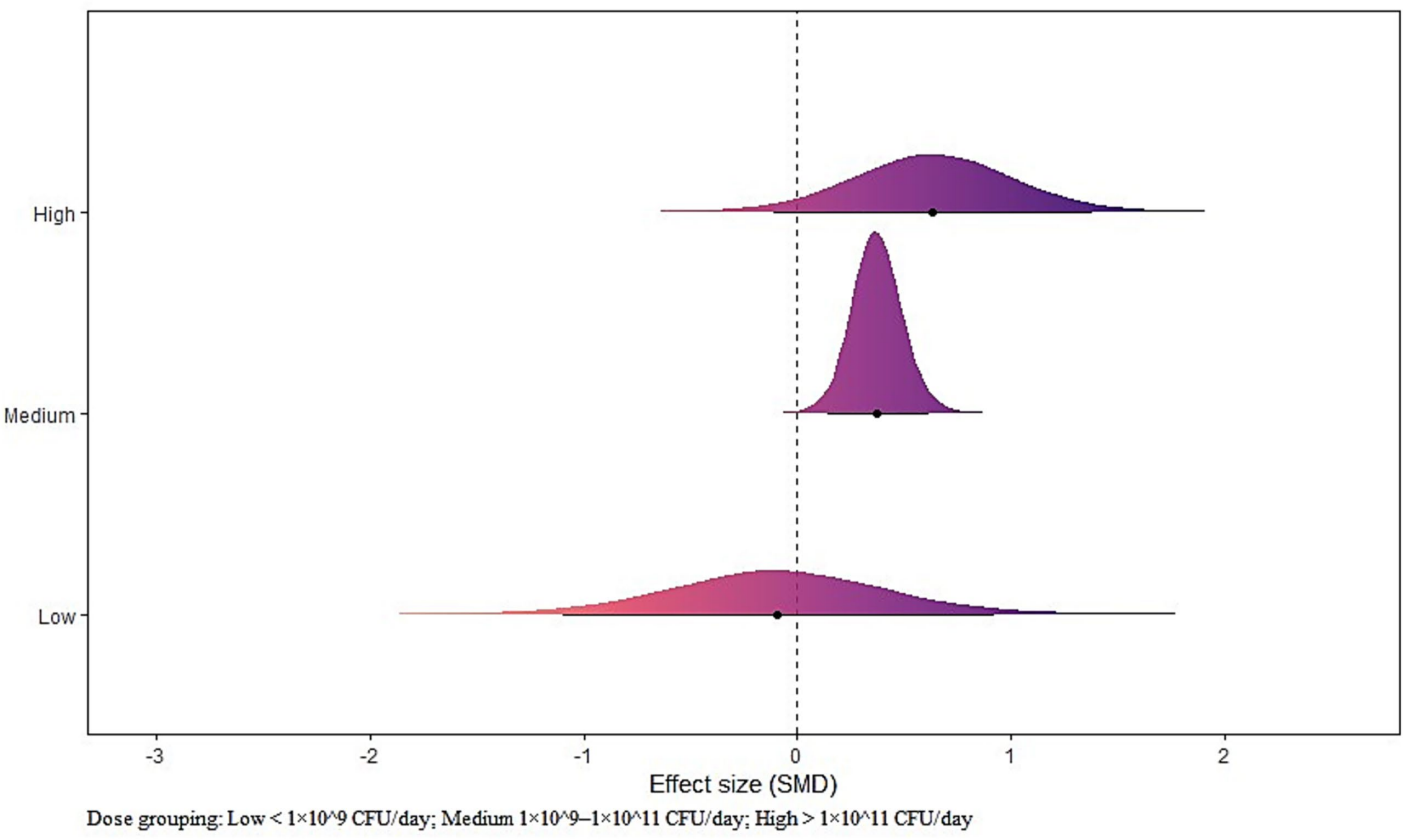
The forest plot in probiotic dosage model.

Medium dose showed a significant effect [μ(SMD): 0.38, 95% CrI: 0.14 to 0.62; HDI: 0.14 to 0.62; BF: 7.44]. Low and High doses were not statistically significant. Heterogeneity was small (*τ*_within_: 0.28; *τ*_between_: 0.28) (see Figure 8).

### Muscle strength model

In the muscle strength model, no statistically significant effect was observed for lower-limb strength [μ(SMD): 0.20, 95% CrI: −0.03 to 0.44; HDI: −0.035 to 0.434] or upper-limb strength [μ(SMD): 0.07, 95% CrI: −0.25 to 0.40; HDI: −0.253 to 0.393]. Across both subdomains, heterogeneity was small (*τ*_within_: 0.13; *τ*_between_: 0.13), with modest decision and across-study inconsistency (DI: 16.6%; ASI: 10.8%). The corresponding forest plot is presented in Supplementary File S5.

### Agility model

In the agility model, no statistically significant effect was observed for either change-of-direction or sprint speed. Heterogeneity was low (*τ*_within_: 0.21; *τ*_between_: 0.21), with modest inconsistency (change-of-direction: DI 12.18%, ASI 5.25%; sprint speed: DI 26.2%, ASI 4.51%). The corresponding forest plot is presented in Supplementary File S5.

### Endurance model

In the endurance model, a statistically significant effect was observed for aerobic endurance [μ(SMD): 0.74, 95% CrI: 0.39 to 1.10; HDI: 0.39 to 1.09; BF: 103.61]. Anaerobic endurance was not statistically significant. Heterogeneity was low across these outcomes (*τ*_within_: 0.22; *τ*_between_: 0.22), and for aerobic endurance the inconsistency metrics were modest (DI: 7.82%; ASI: 54.83%). The corresponding forest plots are presented in Supplementary File S5.

### Lower limb strength model

Only PT60°DsKF reached significance [μ(SMD): −3.64, 95% CrI: −5.15 to −2.11; HDI: −5.16 to −2.13; BF: 1500]. All other lower-limb tests were not statistically significant. Heterogeneity was low (*τ*_within_: 0.18; *τ*_between_: 0.18). The corresponding forest plots are presented in Supplementary File S5.

### Upper-limb strength model

No statistically significant effect was observed for any test (HGS Left Righ Overall, Hand Muscle Strength, 1RM Bench Press, Pull-ups). Heterogeneity was low (*τ*_within_: 0.34; *τ*_between_: 0.34). The corresponding forest plots are presented in Supplementary File S5.

### Anaerobic endurance model

No statistically significant effect across RAST, PPO MPO (cycling), WAnT-30 s, and Continuous Reverse Vertical Jump. Heterogeneity was low (*τ*_within_: 0.34; *τ*_between_: 0.34). The corresponding forest plots are presented in Supplementary File S5.

### Change of direction model

No statistically significant effect (IAT, Ronaldo Speed Test). Heterogeneity was moderate (*τ*_within_: 0.76; *τ*_between_: 0.77). The corresponding forest plots are presented in Supplementary File S5.

### Sprint speed model

No statistically significant effect (10MWT, 40-m Dash, 100-m Sprint, Sprint Test, 10-Yard Sprint). Heterogeneity was low-to-moderate (*τ*_within_: 0.38; *τ*_between_: 0.37). The corresponding forest plots are presented in Supplementary File S5.

### Aerobic endurance model

Significant effects were found for VO₂max (cycle) [μ(SMD): 2.21, 95% CrI: 0.64 to 3.68; HDI: 0.68 to 3.72; BF: 15.41] and RPE (Borg) [μ(SMD): 2.06, 95% CrI: 0.88 to 3.24; HDI: 0.88 to 3.24; BF: 60.21]. Other aerobic endurance outcomes were not statistically significant. Heterogeneity was low (*τ*_within_: 0.30; *τ*_between_: 0.30). The corresponding forest plots are presented in Supplementary File S5.

### Financial support model

A positive estimate was observed for unfunded [μ(SMD): 0.38, 95% CrI: 0.02 to 0.76; HDI: 0.01 to 0.76; BF: 0.847] and funded [μ(SMD): 0.38, 95% CrI: 0.10 to 0.66; HDI: 0.01 to 0.66; BF: 2.44]. Heterogeneity was small (*τ*_within_: 0.15; *τ*_between_: 0.41). The corresponding forest plots are presented in Supplementary File S5.

### Moderation analysis (linear regression)

**Figure 9:**
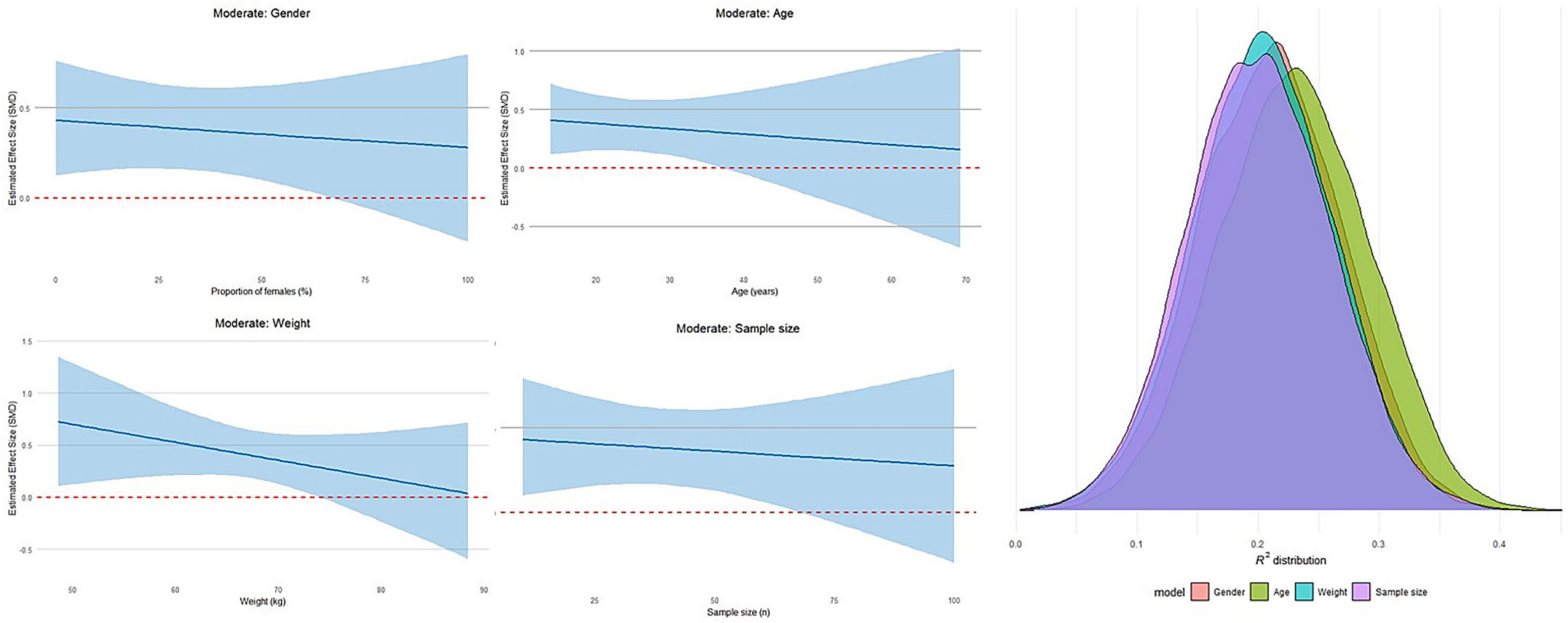
The regression plots and *R*^2^ density plot moderated by age, weight, sample size, and gender in athletic performance.

Four trait moderators were entered into the meta-regression. All moderation plots are shown in Figure 9, and the full numerical outputs are reported in Supplementary File S6. For athletic performance, none of the four moderators gender, age, weight, and sample size showed a statistically significant effect. Across models, explanatory power was small (low *R*^2^), suggesting these traits are unlikely to account for the observed heterogeneity.

### Session duration (moderation)

**Figure 10:**
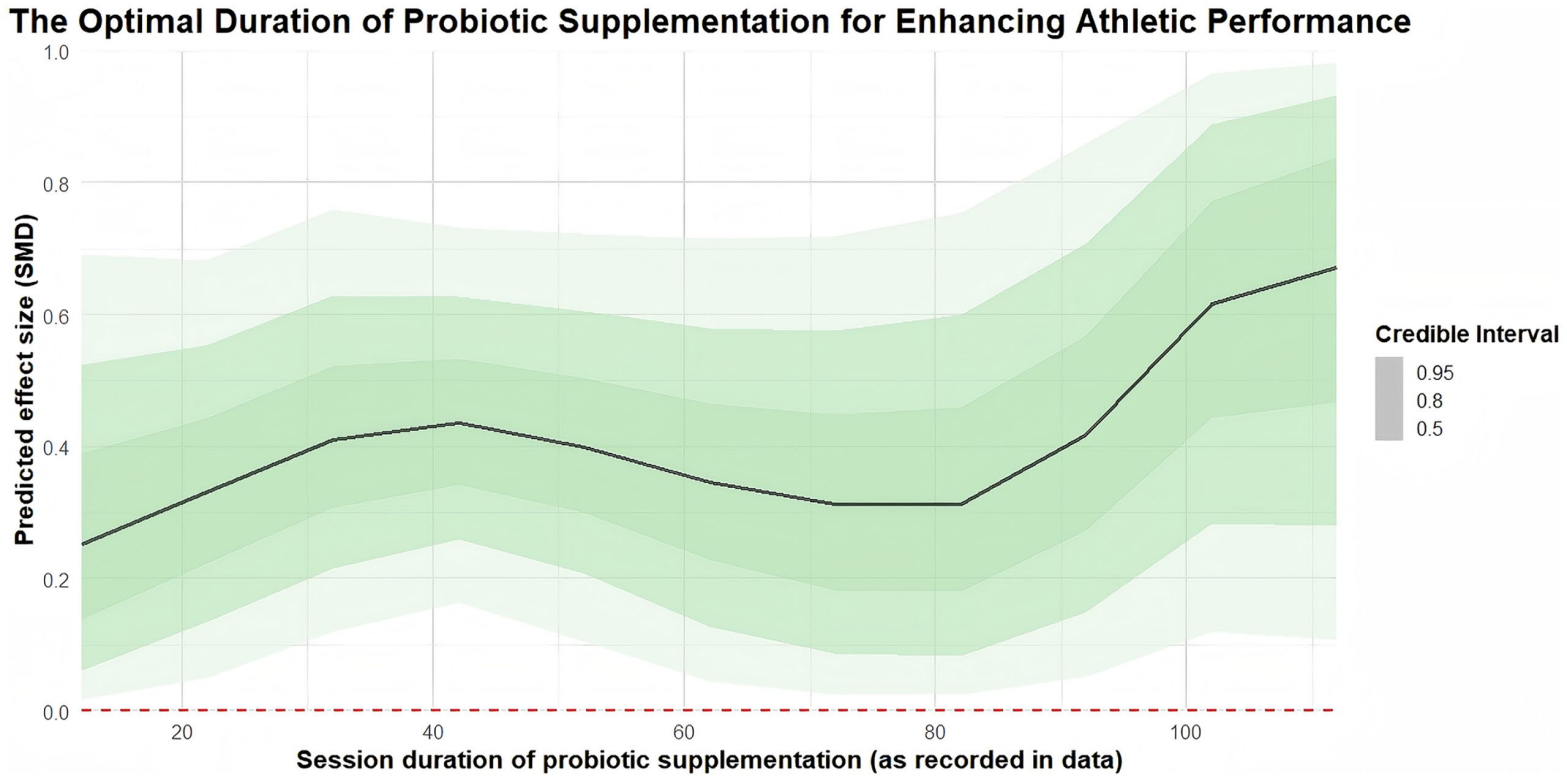
Regression plot of intervention duration moderating athletic performance.

Regarding intervention duration, the results indicate that 110 days yields the greatest improvement in athletic performance (coefficient estimate: 0.93, 95% CrI: 0.24 to 1.64; *R*^2^: 21%), with statistically significant benefits also observed at 30 days (coefficient estimate: 0.41, 95% CrI: 0.14 to 0.72; *R*^2^: 21%), 35 days (coefficient estimate: 0.43, 95% CrI: 0.18 to 0.70; *R*^2^: 21%), and 60 days (coefficient estimate: 0.36, 95% CrI: 0.02 to 0.67; *R*^2^: 21%). The regression plots are presented in Figure 10.

### Quality grade in each outcome

**Figure 11:**
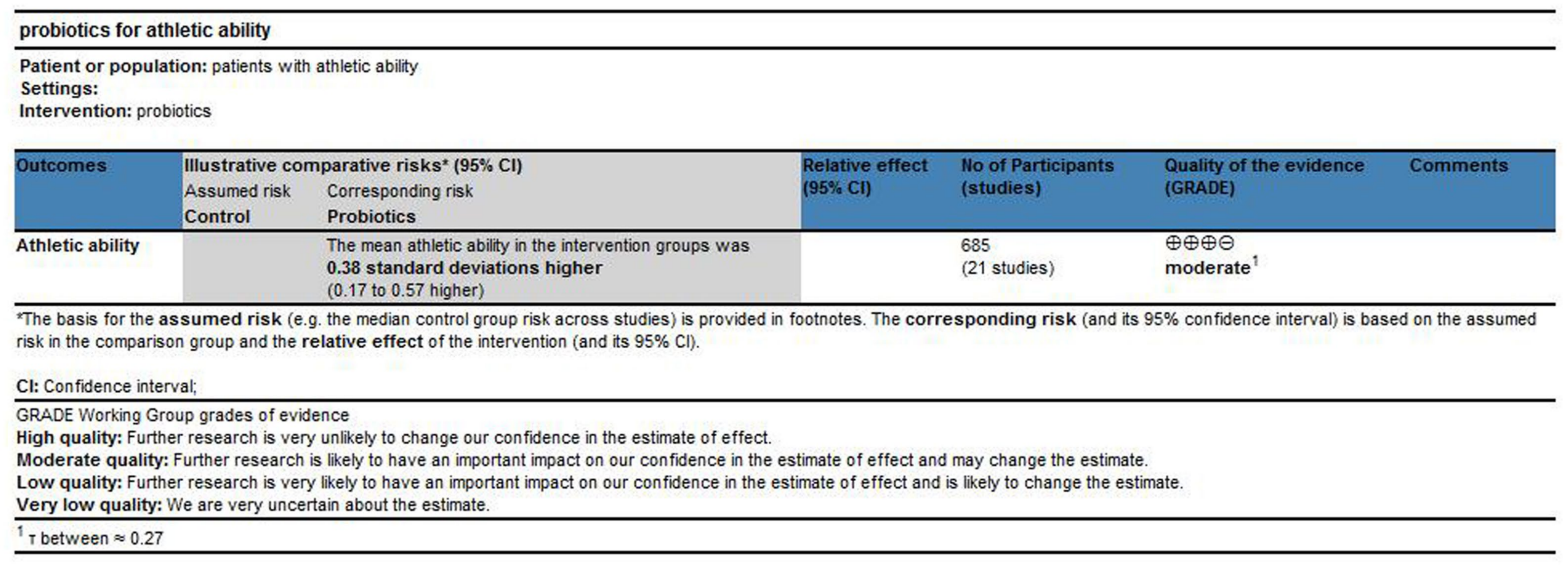
Summary of evidence quality using the GRADE approach.

The certainty of evidence was appraised with GRADE considering sample size, risk of bias, inconsistency, indirectness, and imprecision. Under the null model for athletic ability, the overall certainty was moderate Figure 7. Across subdomains, certainty was moderate for muscle strength and endurance, and low for agility. By dose tier, certainty was low for low dose, moderate for medium dose, and low for high dose. By formulation, both multi-strain probiotics (MSP) and single-strain probiotics (SSP) were rated low. By population, adults were moderate, while athletes and older adults were low. By funding, funded studies were very low and unfunded studies were low. At the strain level, *Lactobacillus plantarum* reached moderate certainty, whereas other reported strains were low. The summary of evidence quality using the GRADE approach is presented in Figure 11. A full set of GRADE tables is provided in Supplementary File S7.

### Publication Bias

**Figure 12:**
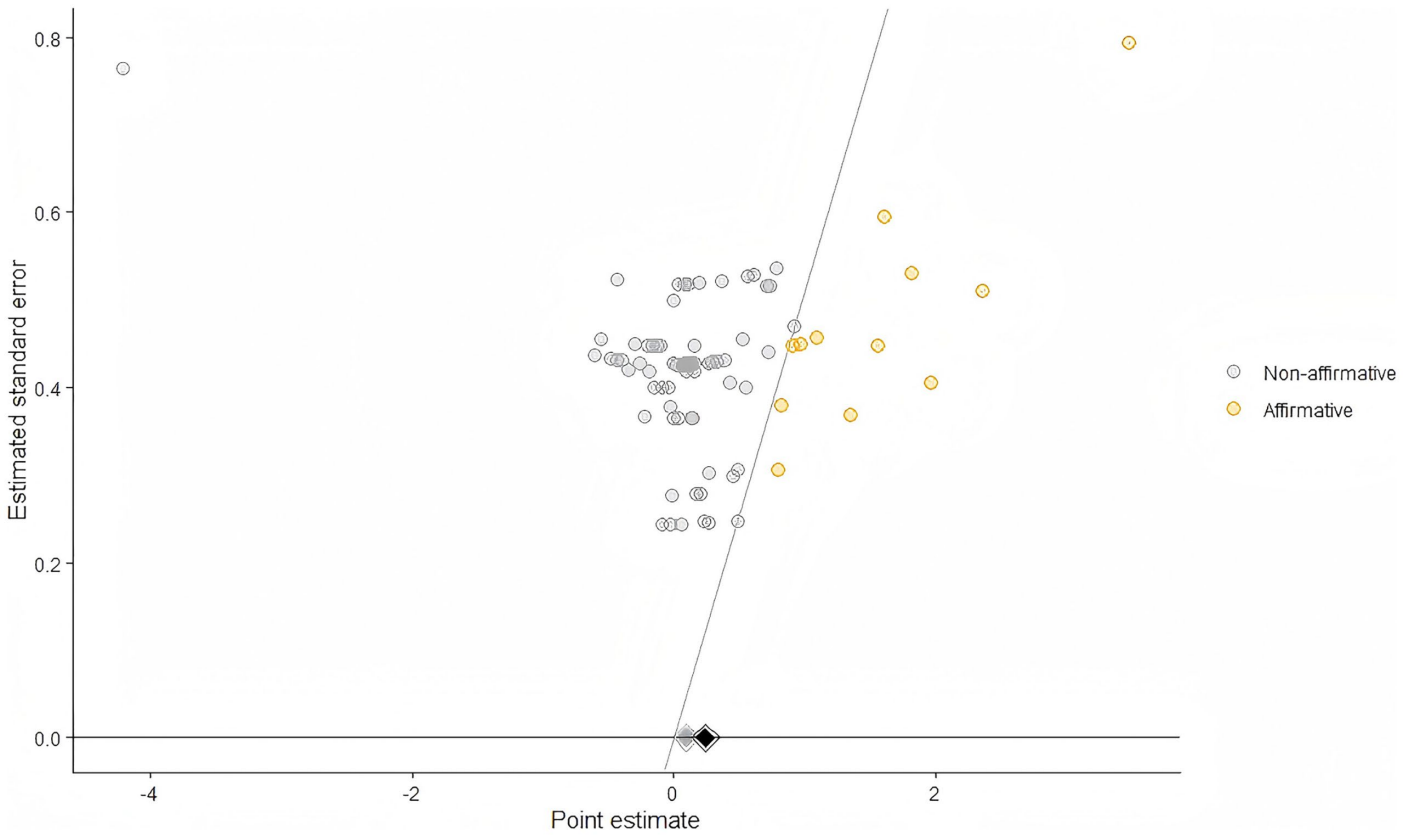
The funnel plot via Publicationbias package (athletic ability).

Figure 8 presents the significance funnel for the overall athletic-ability meta-analysis. First, a multilevel Egger’s test did not indicate publication bias for the overall model (*p* = 0.42). Second, in the significance funnel, most studies fell within non-significant regions; importantly, the grey diamond (non-significant studies only) closely aligned with the black diamond (all studies), suggesting that the overall findings were not materially affected by selective significance. Third, the computed *s*-value indicated that no plausible degree of publication bias would be sufficient to reduce the observed overall effect to the null (“not possible”; interval estimate ≈ 2.03), supporting the robustness of the results. Additionally, a trim-and-fill analysis using metafor did not impute any studies, and no clear asymmetry was observed in the funnel plot, further confirming the stability of the athletic-ability findings. The publication bias (funnel plot) is presented in Figure 12. Other funnel plots (including additional model displays) are provided in Supplementary File S8.

## Discussion

This is the first multilevel meta-analysis to evaluate the efficacy of different probiotic strains, formulation types (single- vs. multi-strain), supplementation duration, added components within probiotic products, and overall daily CFU doses on athletes’ performance. The current systematic review and meta-analysis summarize the evidence on the effect of probiotic supplementation on athletic performance.

### Summary of findings

Across randomized controlled trials synthesized with Bayesian hierarchical models, probiotic supplementation improved athletic performance in healthy individuals, with the clearest and most consistent gains in endurance-centric outcomes (e.g., VO₂max time-to-exhaustion). Benefits were evident in athlete and adults populations, while inference for older adults was limited by sparse data. Both single-strain (SSP) and multi-strain (MSP) regimens were effective, with multi-strain formulations seeming to yield larger improvements; Medium doses (1 × 10^9^–1 × 10^11^ CFU·day^−1^) captured the most reliable signal in subgrouping. Moderator analyses of duration indicated a nonlinear pattern: by about 30 days a small to moderate improvement in performance was evident, and by about 90 days the effect reached the largest predicted gain.

### Effects of probiotic intake on athletic ability

Across randomized trials synthesized in this review, probiotic supplementation was associated with a small to moderate improvement in overall athletic performance, with the clearest and most consistent gains in endurance outcomes. This pattern appeared in both athletes and non-athlete adults and was observed with single-strain and multi-strain products.

Multiple randomized trials and evidence syntheses support these findings, consistently highlighting endurance-dominant benefits and health-mediated training continuity with probiotic use (1, 3, 12, 16, 20). Athlete-focused position statements and syntheses consistently report lower incidence and shorter duration of upper respiratory tract infections and gastrointestinal complaints with selected strains, helping maintain training continuity and competition readiness (1). Trials in athletes echo this health-to-performance pathway (55), and a randomized trial in nonelite runners prospectively targeted reductions in gastrointestinal and cold-flu symptoms to facilitate training progression, reporting endurance benefits after several weeks of use (55). Reviews converge that benefits are strain and context dependent, and that endurance outcomes respond more reliably than strength or speed in short interventions (12). Evidence in older adults is currently insufficient in our dataset (42, 56).

Within a gut muscle axis framework, probiotics can increase short-chain fatty acid availability and signaling, modulate immune and neuroendocrine pathways, improve mitochondrial function and substrate utilization, strengthen epithelial and mucosal barrier integrity, and temper exercise-induced inflammation and oxidative stress a constellation that plausibly underpins the endurance-dominant gains we observed (57). In older populations, mechanistic work suggests short-chain fatty acids may mitigate low-grade inflammation and support muscle metabolism, motivating longer-duration trials with endurance-relevant functional endpoints (57, 58).

Endurance benefits are consistent with randomized trials using *Lactobacillus plantarum* strains such as TWK10, which report improvements in VO₂-related endurance and time-to-exhaustion together with favorable shifts in fatigue biomarkers and body composition (3, 16, 20). Strength findings were less consistent overall; short interventions may be too brief to capture neuromuscular remodeling and hypertrophy timelines, and current expert guidance notes that many benefits in athletes are indirect (immunity and gastrointestinal health) rather than directly ergogenic for peak force (21, 59). Evidence for agility and sprint remains mixed and sparse; heterogeneity in task metrics and brief exposures likely dilute detection power, underscoring the need for task standardization and adequate intervention duration (60, 61).

Our dose-tier analysis indicated that the Medium tier (1 × 10^9^ to 1 × 10^11^ CFU per day) produced the most reliable improvement. This is broadly compatible with sports-nutrition guidance and recent syntheses that emphasise adequate daily exposure for several weeks at or above 10^9^ CFU per day, particularly for endurance-relevant outcomes (1, 12). At the same time, variability in how trials report dose (units, viable counts by delivery form) complicates formal dose–response modelling across studies (12). Notably, many randomised trials and evidence syntheses that reported endurance- or strength-relevant benefits used daily doses within this Medium window: *Lactiplantibacillus plantarum* TWK10 at approximately 3 × 10^10^ to 9 × 10^10^ CFU per day over 6 weeks improved time-to-exhaustion (3); *Lactobacillus plantarum* PL-02 at about 1.5 × 10^10^ CFU per day for 6 weeks improved muscle strength and endurance (43); *Lactococcus lactis* LY-66 over a comparable six-week schedule enhanced endurance and explosive strength (9); and endurance-focused syntheses aggregating athlete trials likewise concentrate effective exposures in the 10^9^–10^11^ CFU per day band (16, 20). Together, these patterns support the Medium tier as a practical starting point, with strain selection and sustained duration tailored to the target outcome (1).

Single-strain and multi-strain products were both effective in our models. Athlete-oriented reviews often favor multi-strain when the aim is to reduce gastrointestinal complaints and upper-respiratory illness burden to preserve training continuity (1, 62). Multi-strain mixes may offer advantages over single-strain in this health maintenance context, insofar as complementary functions across strains are hypothesized to broaden metabolic and immune coverage, ecological redundancy could buffer inter-individual microbiota variability, and a wider adhesion profile might support barrier integrity and day-to-day gastrointestinal comfort under heavy training factors that together may increase the likelihood of benefit at a given daily dose and exposure period (12, 63). Consequently, multi-strain is often the pragmatic default for in-season support (1, 62). By contrast, single-strain formulations are preferred where a validated strain-to-outcome link exists for the specific performance target, as shown for *Lactobacillus plantarum* TWK10 in endurance settings (3, 16, 20). Effects remain strain and context dependent and hinge on formulation quality, viable counts, and sufficient duration (12).

### Heterogeneity in moderation analyses and its potential sources

We observed moderate heterogeneity concentrated in endurance and its subdomains (lower limb endurance, VO₂max, fatigue). Differences in exercise tests platforms, calibration, VO₂max computation, and fatigue indices likely inflated between-study variance, a pattern noted in endurance–microbiome work (64). Even after domain stratification, outcome definitions and protocols were not fully harmonized (e.g., treadmill vs. cycle-ergometry VO₂max; distinct speed/agility tests), sustaining moderate inconsistency in some submodels (65–67). Many included RCTs also featured small samples and brief exposures, constraining precision and external validity (8, 19, 41, 44, 53).

Accordingly, future trials should prioritize larger, multicentre, adequately blinded designs with dose-graded arms and standardized outcome batteries to improve estimate stability and generalizability (68). Although we examined daily dose by prespecified tiers and compared SSP/MSP formulations, a unified dose–response meta-regression was not pursued given these methodological constraints. Current guidance likewise emphasizes strain-specific recommendations and sustained exposure rather than a single universal daily dose; dose-ranging RCTs are needed to define optimal CFU windows by strain and performance domain (60, 63, 69, 70).

We also examined intervention duration as a separate moderator. Several exposure windows are plausible on biological and training grounds. About 30–35 days aligns with a typical mesocycle, during which reductions in upper-respiratory and gastrointestinal symptoms and early short-chain-fatty-acid-linked signalling may support training continuity and performance on validated tests in adult endurance contexts (1, 14). Around ~60 days allows further microbiota remodelling, substrate use, barrier function, and immune tone to develop—patterns consistent with trials using sport-characterized strains such as *Lactobacillus plantarum* in endurance settings (3, 16, 20). Extending to ~3–4 months permits adaptation across multiple mesocycles with fewer illness interruptions and steadier gastrointestinal tolerance consistent with mechanistic reviews highlighting mitochondrial and inflammatory pathways relevant to endurance performance (1, 57).

### Strengths and limitations

This is the first Bayesian multilevel meta-analysis to investigate probiotic effects on athletic ability. This review was prospectively registered and reported in accordance with PRISMA, with two-reviewer screening, extraction and risk-of-bias assessment, ensuring procedural transparency and replicability (22). Methodologically, we synthesized effects via Bayesian multilevel models (brms) with random effects for within- and between-study variance, and complemented traditional heterogeneity with dissimilarity index (DI) and across-studies inconsistency (ASI) to appraise inconsistency against decision thresholds an approach aligned with best practice for Bayesian meta-analysis (35). Beyond Bayesian synthesis, we implemented cross-paradigm publication bias checks standard funnel/Egger and trim and fill alongside significance-based diagnostics, mirroring prior work’s combined Frequentist–Bayesian bias assessment (33). Screening leveraged active-learning assisted workflows (ASReview with SAFE rule) and modern conversion/visualization utilities to reduce manual burden while maintaining auditability, consistent with contemporary systematic-review pipelines (23, 24). Finally, we graded the certainty of evidence using GRADE across overall and stratified models (formulation SSP/MSP; population), providing decision-relevant confidence statements rather than *p*-values alone (35).

However, despite these strengths, several limitations remain. Even after domain stratification, outcome definitions and test protocols were not fully harmonized, limiting direct comparability and sustaining moderate inconsistency across stratified models. Many included RCTs featured small samples and short intervention periods, constraining precision and external validity; long-term follow-up was rare, consistent with prior evidence syntheses that rated overall certainty downwards due to risk of bias, heterogeneity, imprecision and potential small-study effects. Although we conducted SSP/MSP subgroup analyses, a unified dose–response meta-regression was not undertaken due to heterogeneous dose reporting (CFU units, viable counts per vehicle) and variable exposure durations. While GRADE tables contextualize effects by domain, dose and formulation, several strata remained at low/very-low certainty, indicating that estimates should be interpreted cautiously and that larger, adequately blinded, multi-centre trials with dose-graded arms and uniform outcome batteries are needed to improve estimate stability and generalizability.

## Conclusion

In this preregistered systematic review and Bayesian meta-analysis of randomized trials, probiotic supplementation produced a small-to-moderate, practically meaningful improvement in athletic performance among healthy individuals, with the clearest and most consistent gains in endurance-centric outcomes (e.g., VO₂max and time-to-exhaustion). Benefits were observed with both single-strain and multi-strain formulations, and medium daily doses (~10^9^–10^11^ CFU) yielded the most reliable signal findings that align with biologically plausible mechanisms along the gut brain muscle axis (barrier integrity, immune modulation, short-chain fatty acids, and substrate utilization). Publication-bias diagnostics and GRADE ratings at the domain level support confidence in the direction of effect while acknowledging variability across tests and subgroups.

At the same time, the current evidence base is limited by small samples, short intervention periods, heterogeneous dose reporting, and non-standardized outcome batteries, which constrain precision and generalizability. To translate these probabilistic gains into practice-ready guidance suited for sports nutrition, future research should prioritize multi centre, adequately blinded, dose-ranging trials with faithful strain reporting (identity and viable counts), harmonized endurance and strength test protocols, and prespecified moderator analyses. Such trials will enable strain-resolved and dose-responsive recommendations for athletes and physically active adults and clarify whether longer exposures or targeted strains can extend benefits beyond endurance to strength and agility outcomes. Overall, probiotics constitute a feasible, non-pharmacological adjunct within an evidence-based performance strategy—most convincingly for endurance at present pending confirmatory trials that refine the who, what, and how much.

## Data Availability

The original contributions presented in the study are included in the article/supplementary material, further inquiries can be directed to the corresponding author.
