# Supplementary material for: Effect of probiotic intake on athletic ability in healthy people: a systematic review and Bayesian meta-analysis
Source: Front Nutr. 2026 Jan 30;13:1731627. doi: 10.3389/fnut.2026.1731627 (PMC12903275; doi:10.3389/fnut.2026.1731627)
Supplement: Supplementary file 1 [file Data_Sheet_1.zip › Supplementary file S7 GRADE plots.docx]

**Supplementary File S7: GRADE Summary**


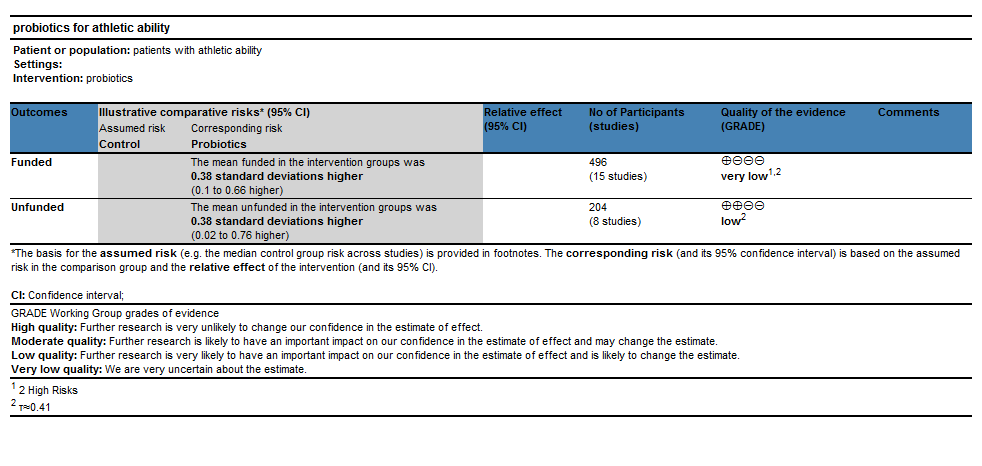


**Figure 1.** The GRADE Summary in Financial support model


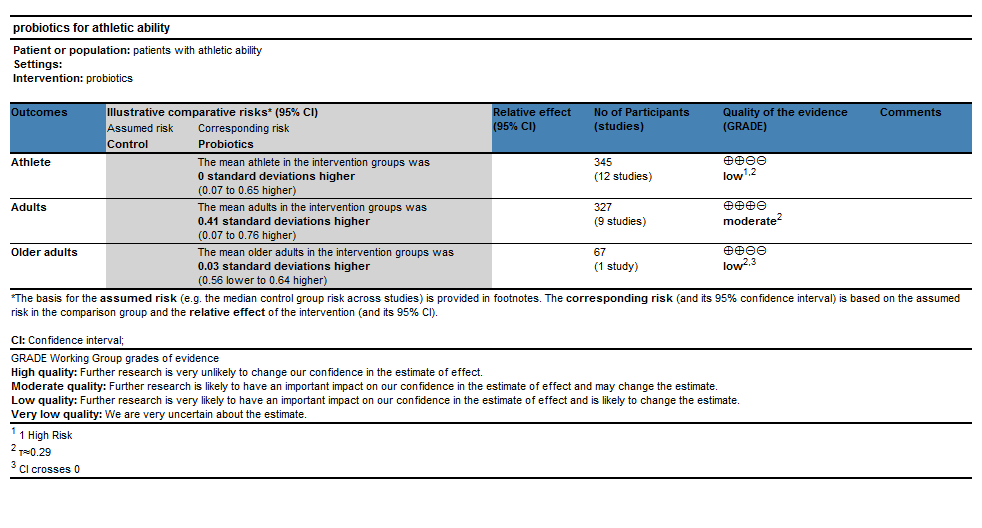


**Figure 2.** The GRADE Summary in Demographic model


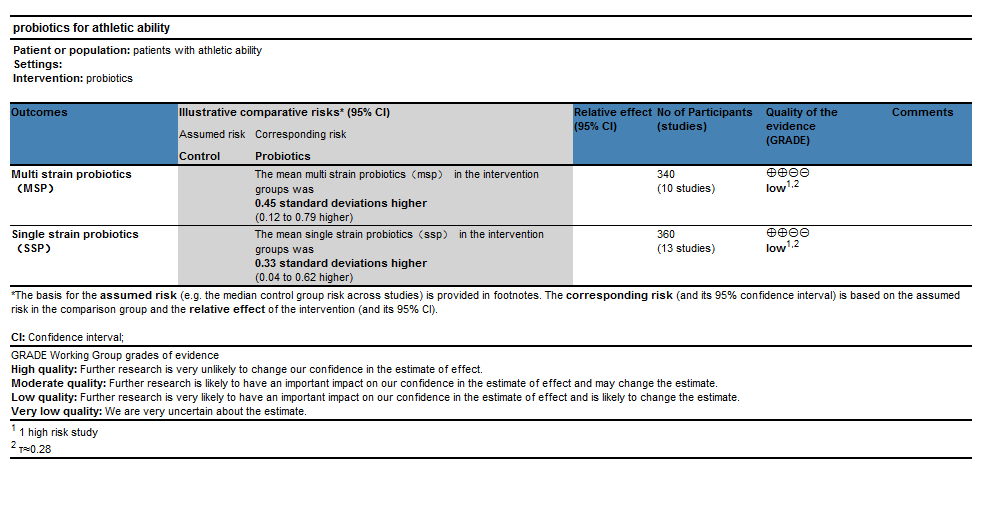


**Figure 3.** The GRADE Summary in Probiotic formulation type model


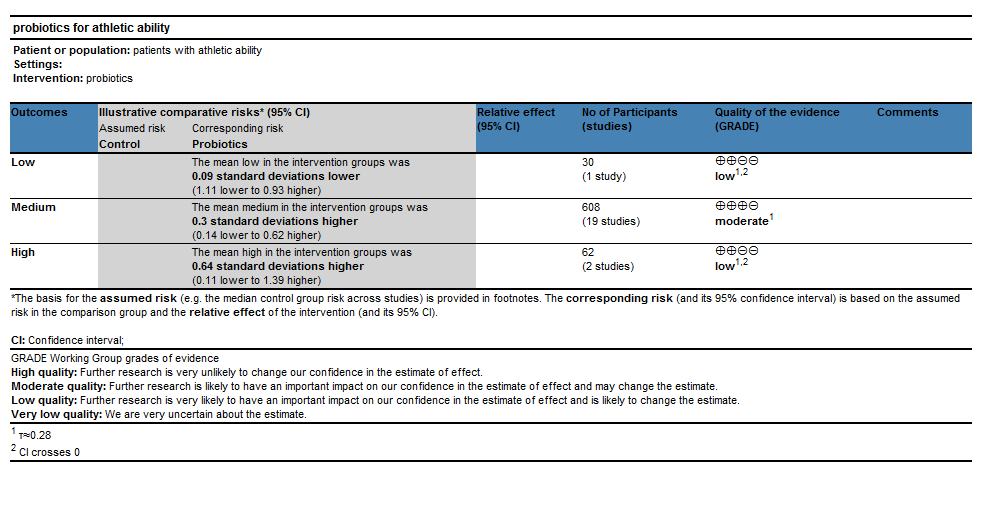


**Figure 4.** The GRADE Summary in Probiotic dosage model


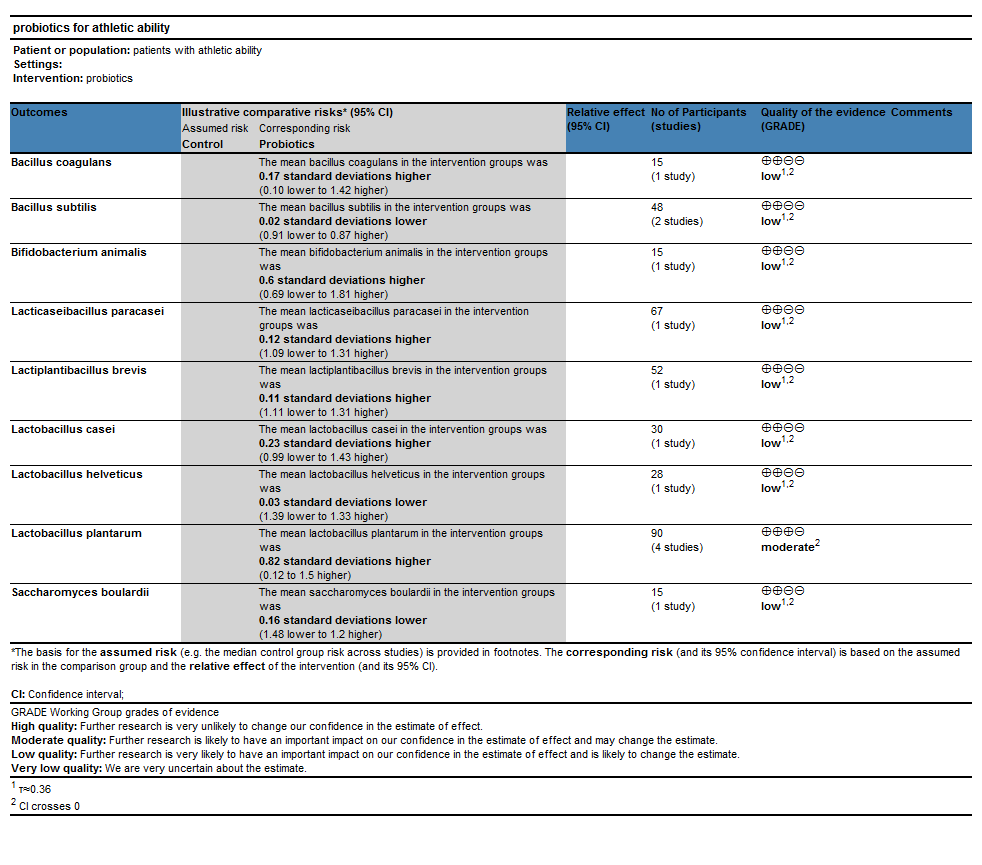


**Figure 5.** The GRADE Summary in Single strain model
