# Supplementary material for: Effect of probiotic intake on athletic ability in healthy people: a systematic review and Bayesian meta-analysis
Source: Front Nutr. 2026 Jan 30;13:1731627. doi: 10.3389/fnut.2026.1731627 (PMC12903275; doi:10.3389/fnut.2026.1731627)
Supplement: Supplementary file 1 [file Data_Sheet_1.zip › Supplementary file S8 Funnel plots.docx]

**Supplementary File S10: Funnel Plots**


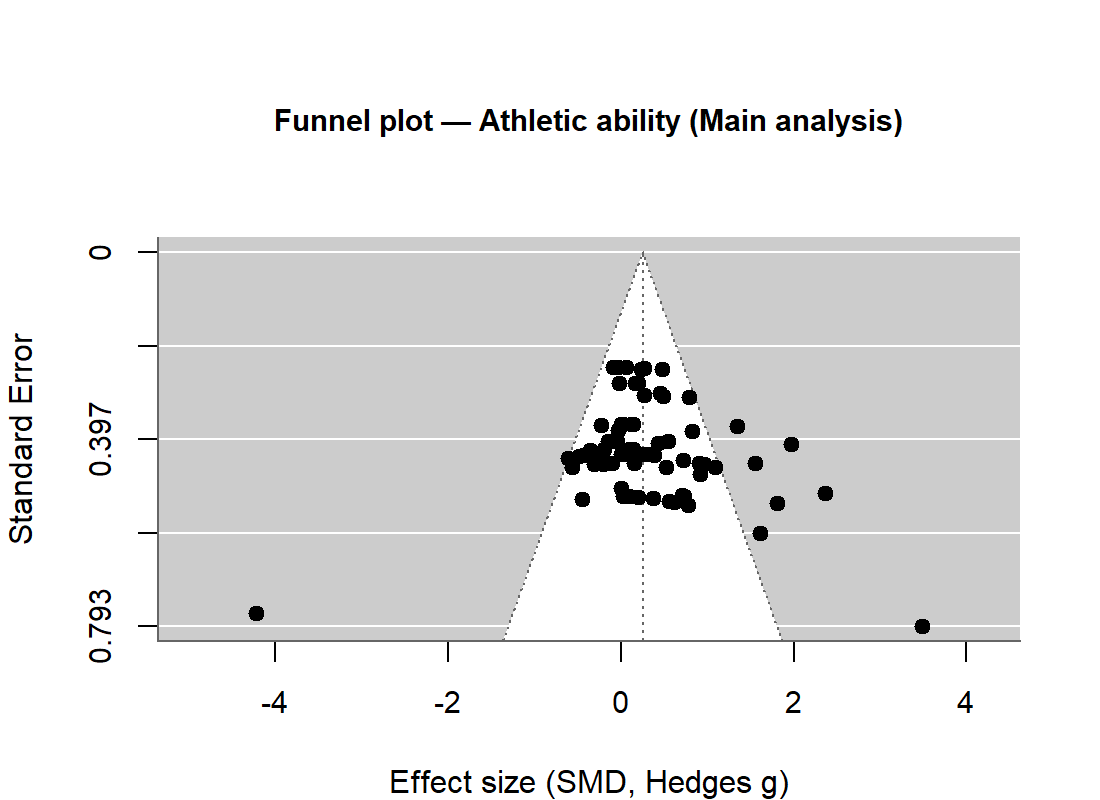


**Figure 1.** The Funnel Plot via Metafor Package (Athletic ability)


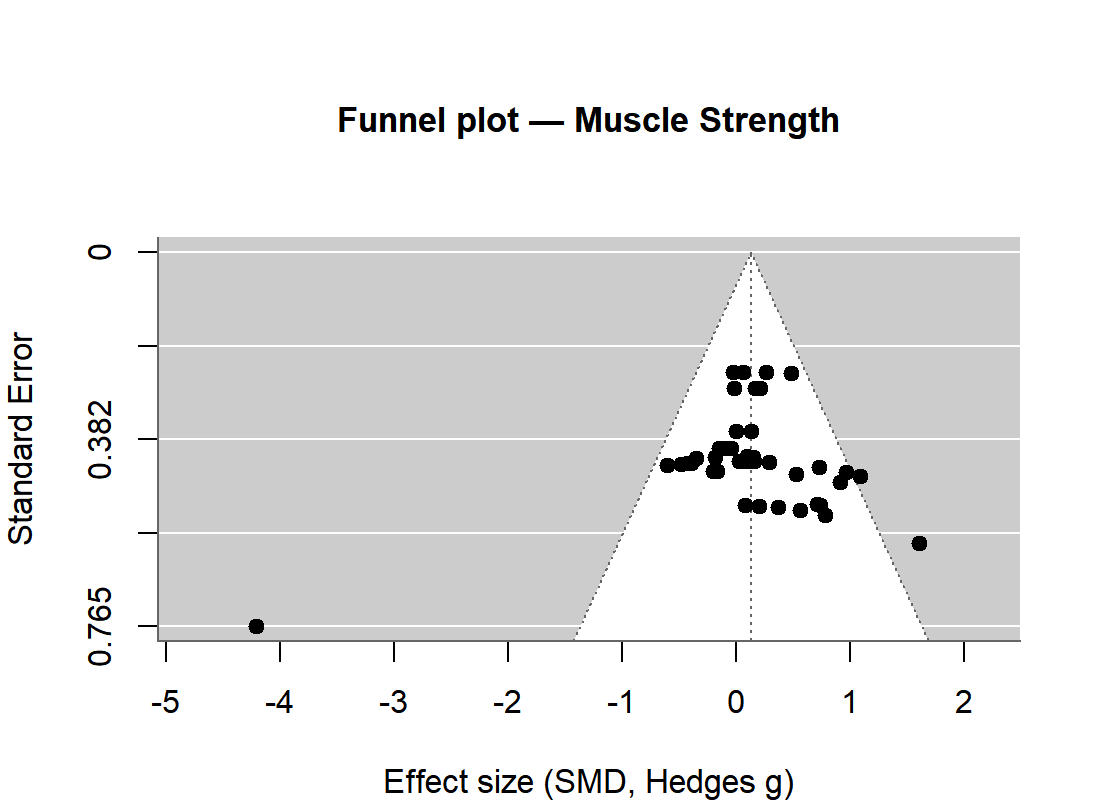


**Figure 2.** The Funnel Plot via Metafor Package (Muscle strength)


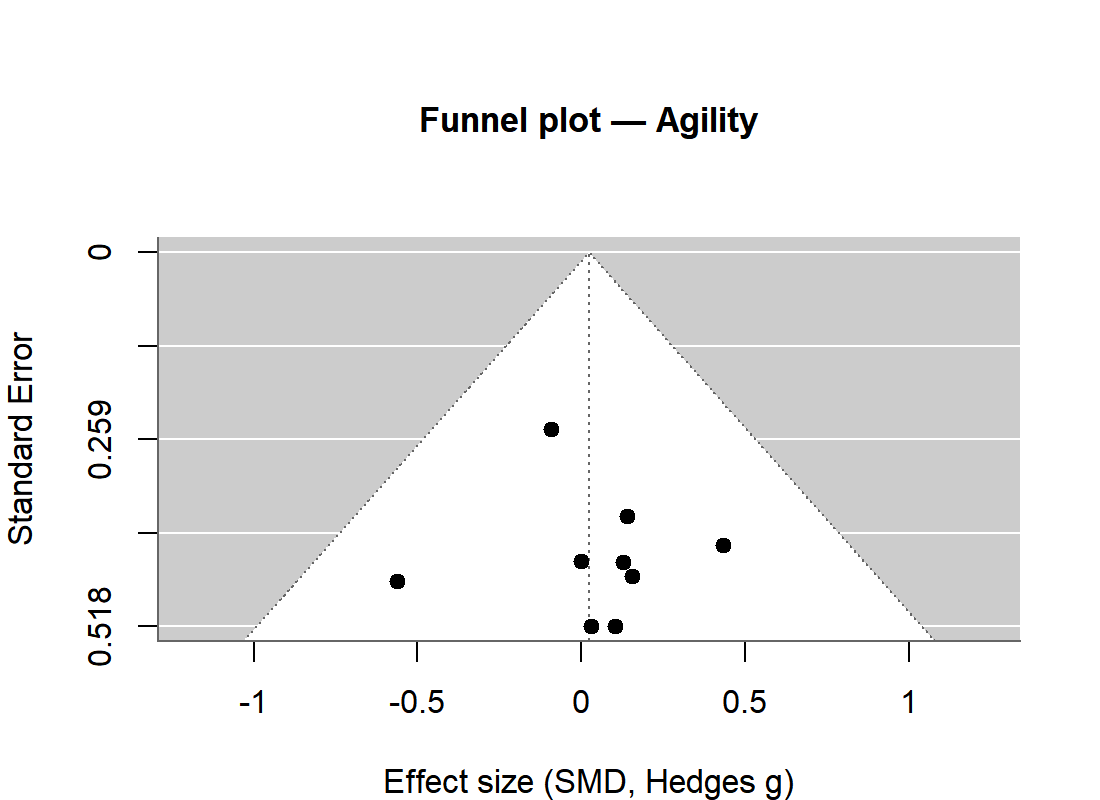


**Figure 3.** The Funnel Plot via Metafor Package (Agility)


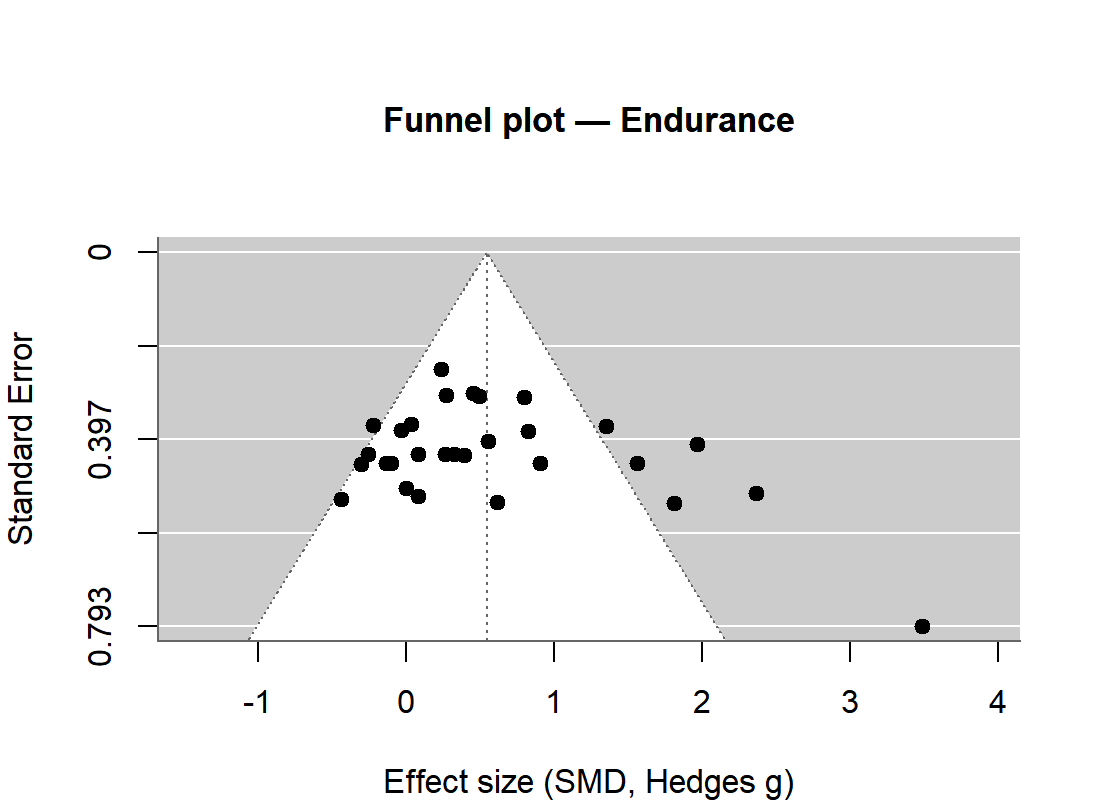


**Figure 4.** The Funnel Plot via Metafor Package (Endurance)


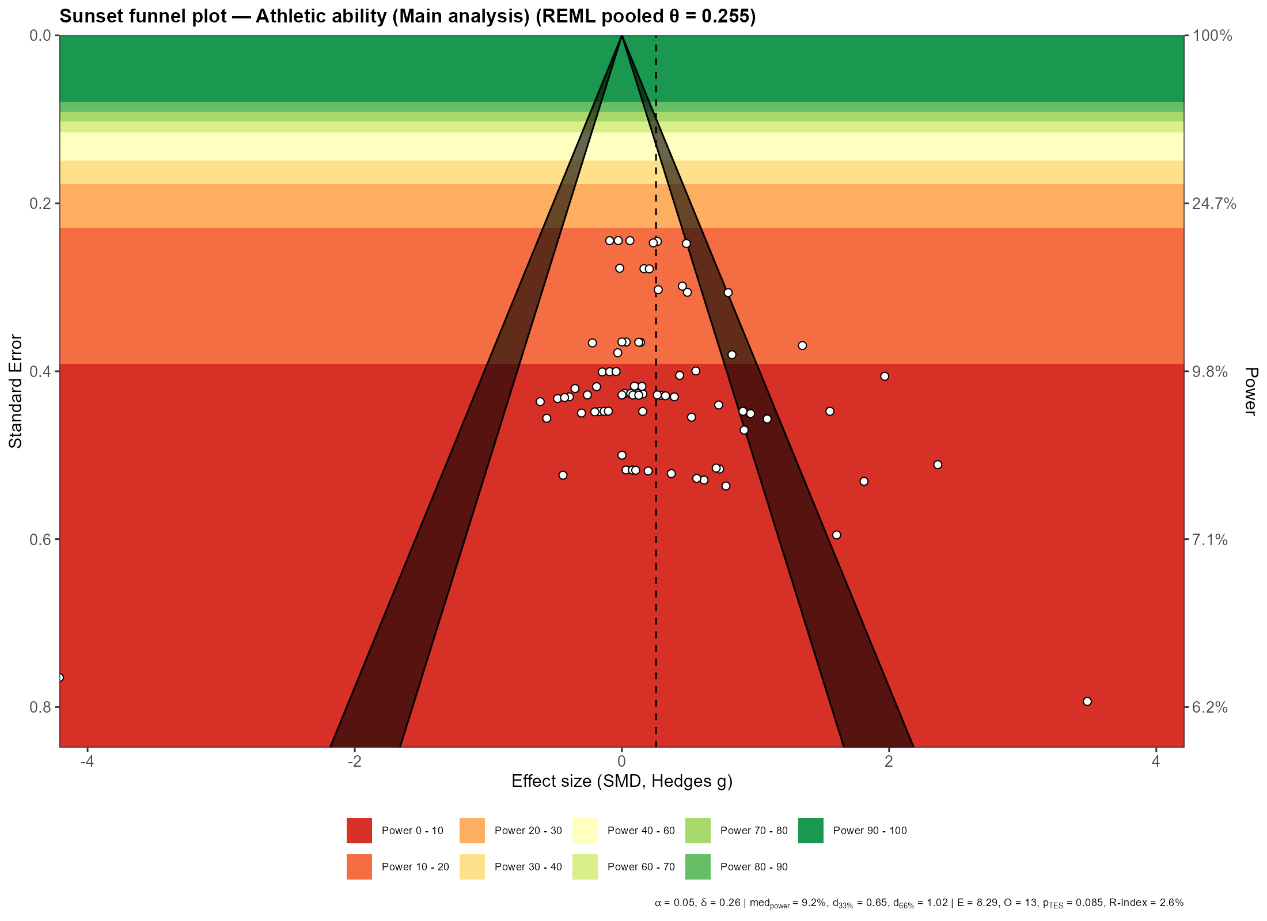


**Figure 5.** The funnel plot for Athletic ability Sunset (power enhanced)


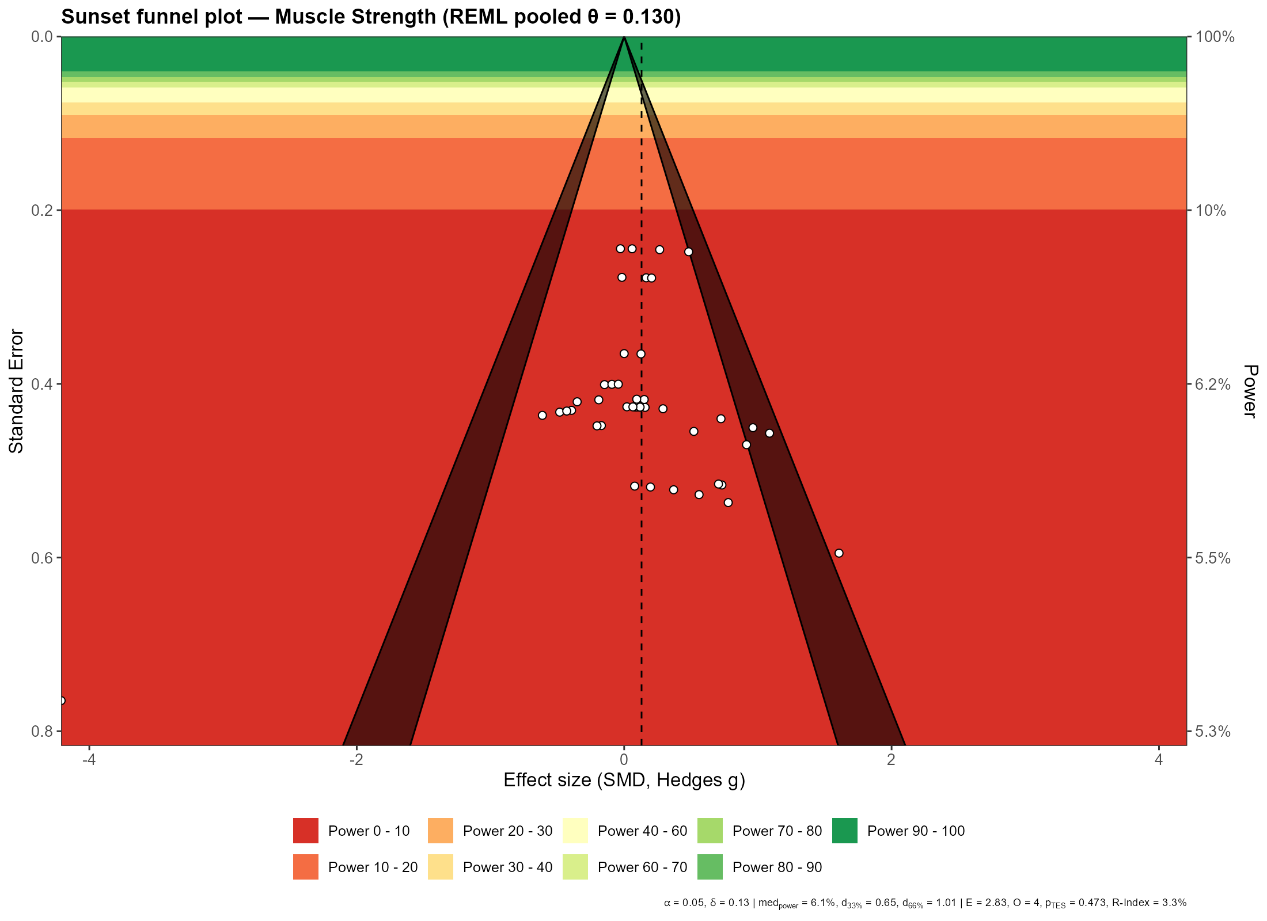


**Figure 6.** The funnel plot for athletic Muscle strength Sunset (power enhanced)


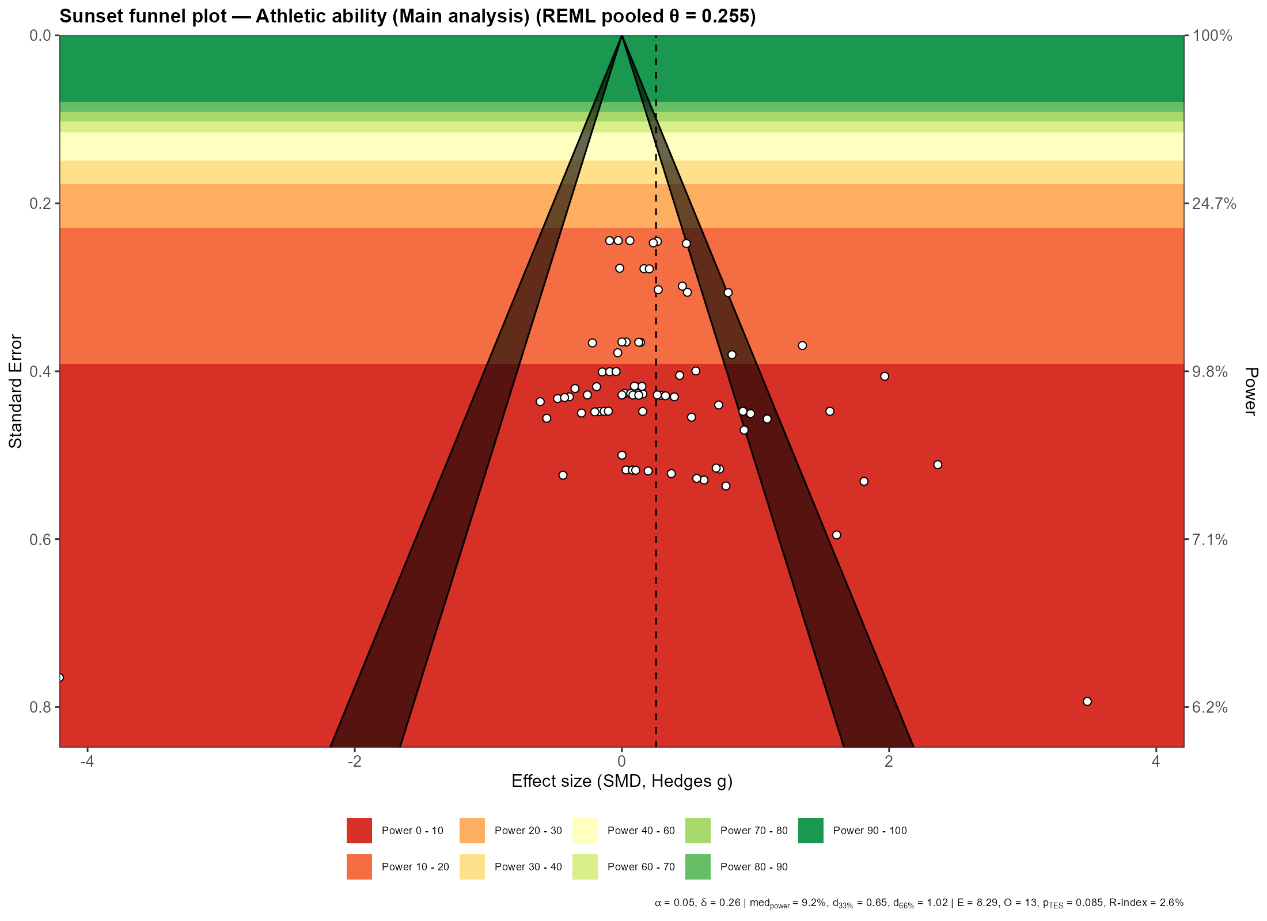


**Figure 7.** The funnel plot for Agility Sunset (power enhanced)


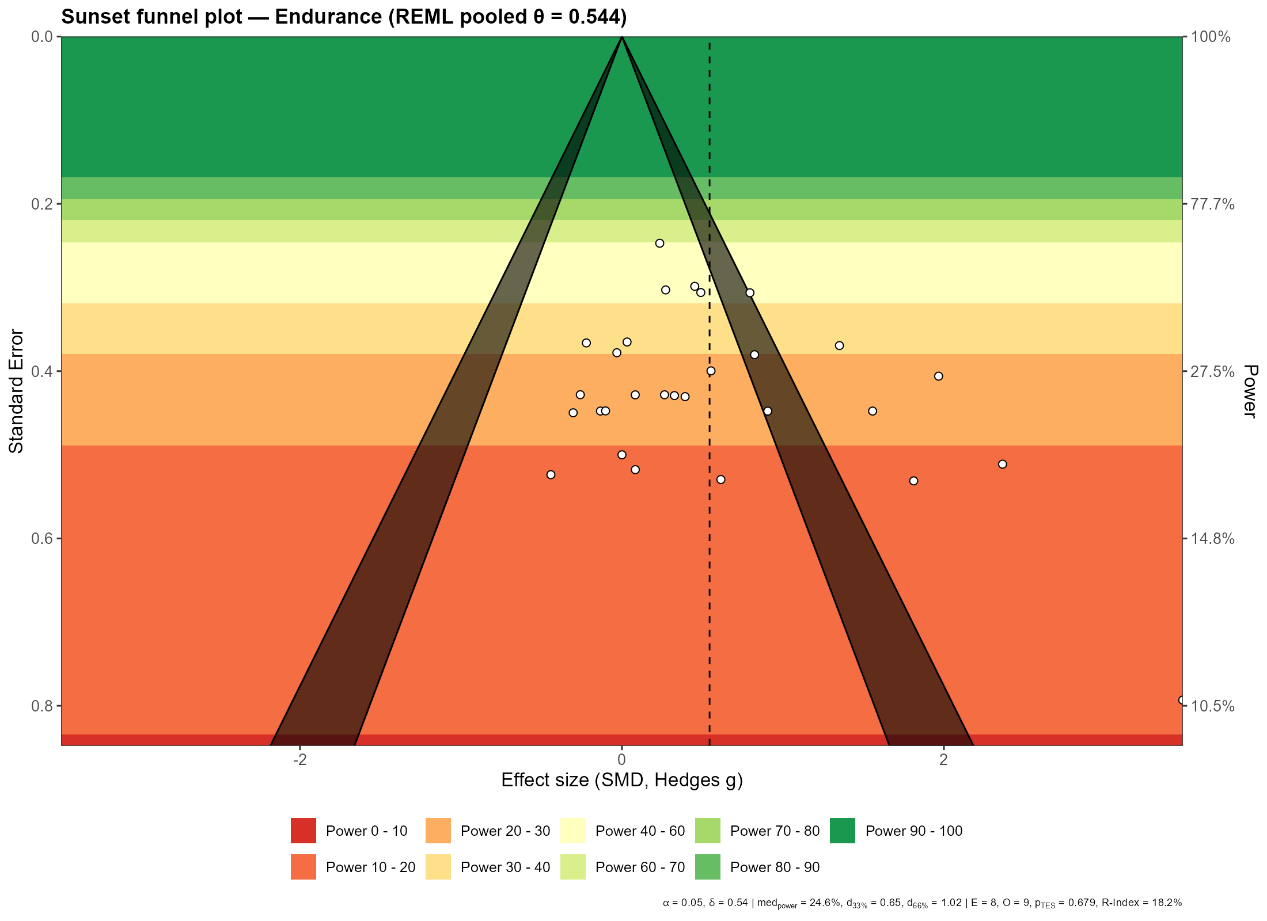


**Figure 8.** The funnel plot for Endurance Sunset (power enhanced)
