# Supplementary material for: Effect of probiotic intake on athletic ability in healthy people: a systematic review and Bayesian meta-analysis
Source: Front Nutr. 2026 Jan 30;13:1731627. doi: 10.3389/fnut.2026.1731627 (PMC12903275; doi:10.3389/fnut.2026.1731627)
Supplement: Supplementary file 1 [file Data_Sheet_1.zip › Supplementary file S10 PRISMA checklist.docx]

| **Section and Topic** | **Item #** | **Checklist item** | **Location where item is reported** |
| --- | --- | --- | --- |
| **TITLE** | | |  |
| Title | 1 | Identify the report as a systematic review. | √  Title page |
| **ABSTRACT** | | |  |
| Abstract | 2 | See the PRISMA 2020 for Abstracts checklist. | √page 1 |
| **INTRODUCTION** | | |  |
| Rationale | 3 | Describe the rationale for the review in the context of existing knowledge. | √  Pages 1 to 3 |
| Objectives | 4 | The objectives and three hypotheses are explicitly stated, outlining expected effects, optimal dose, and strain/formulation expectations. | √  Page 3 |
| **METHODS** | | |  |
| Eligibility criteria | 5 | Specify the inclusion and exclusion criteria for the review and how studies were grouped for the syntheses. | √  Pages 4 |
| Information sources | 6 | Specify all databases, registers, websites, organisations, reference lists and other sources searched or consulted to identify studies. Specify the date when each source was last searched or consulted. | √  Page 4 and 5 |
| Search strategy | 7 | Present the full search strategies for all databases, registers and websites, including any filters and limits used. | √  Supplementary file S1 |
| Selection process | 8 | Screening procedures, including use of ASReview and Covidence, and independent double review with conflict resolution are described. | √  Page 5 |
| Data collection process | 9 | Specify the methods used to collect data from reports, including how many reviewers collected data from each report, whether they worked independently, any processes for obtaining or confirming data from study investigators, and if applicable, details of automation tools used in the process. | √  Page 5 and 6 |
| Data items | 10a | All primary and secondary performance outcomes (e.g., VO₂max, endurance, strength) are listed and defined. | √  Page 5 and 6 |
|  | 10b | Additional variables such as age, sex, probiotic dose, formulation type, and assumptions for missing data (e.g., r = 0.5) are described. | √  Page 5 and 6 |
| Study risk of bias assessment | 11 | Specify the methods used to assess risk of bias in the included studies, including details of the tool(s) used, how many reviewers assessed each study and whether they worked independently, and if applicable, details of automation tools used in the process. | √  Page 6 |
| Effect measures | 12 | Specify for each outcome the effect measure(s) (e.g. risk ratio, mean difference) used in the synthesis or presentation of results. | √  Page 7 and 8 |
| Synthesis methods | 13a | Procedures for determining study eligibility for each synthesis are described. | √  Page 7 and 8 |
|  | 13b | Methods for handling missing data, calculating effect sizes, and data transformations are described. | √  Page 5 and 6 |
|  | 13c | Results are tabulated and visualized using forest plots and density plots. | √  Page 14 to 16 |
|  | 13d | Bayesian multilevel meta-analyses using brms are described, including heterogeneity metrics (τ², I², DI, ASI) and software used (R). | √  Page 7 to 9 |
|  | 13e | Describe any methods used to explore possible causes of heterogeneity among study results (e.g. subgroup analysis, meta-regression). | √  Page 8 and 9 |
|  | 13f | Describe any sensitivity analyses conducted to assess robustness of the synthesized results. | √  Page 8 and 9 |
| Reporting bias assessment | 14 | Describe any methods used to assess risk of bias due to missing results in a synthesis (arising from reporting biases). | √  Page 5 |
| Certainty assessment | 15 | Describe any methods used to assess certainty (or confidence) in the body of evidence for an outcome. | √  Page 6 and 7 |
| **RESULTS** | | |  |
| Study selection | 16a | Describe the results of the search and selection process, from the number of records identified in the search to the number of studies included in the review, ideally using a flow diagram. | √  Page 11 |
|  | 16b | Cite studies that might appear to meet the inclusion criteria, but which were excluded, and explain why they were excluded. | √  Page 11 |
| Study characteristics | 17 | Cite each included study and present its characteristics. | √  Page 11 |
| Risk of bias in studies | 18 | Present assessments of risk of bias for each included study. | √  Page 13 and 14 |
| Results of individual studies | 19 | For all outcomes, present, for each study: (a) summary statistics for each group (where appropriate) and (b) an effect estimate and its precision (e.g. confidence/credible interval), ideally using structured tables or plots. | √  Page 13 to 19 |
| Results of syntheses | 20a | For each synthesis, briefly summarise the characteristics and risk of bias among contributing studies. | √  Page 13 to 19 |
|  | 20b | Present results of all statistical syntheses conducted. If meta-analysis was done, present for each the summary estimate and its precision (e.g. confidence/credible interval) and measures of statistical heterogeneity. If comparing groups, describe the direction of the effect. | √  Page 13 to 19 |
|  | 20c | Present results of all investigations of possible causes of heterogeneity among study results. | √  Page 19 to 21 |
|  | 20d | Present results of all sensitivity analyses conducted to assess the robustness of the synthesized results. | √ |
| Reporting biases | 21 | Present assessments of risk of bias due to missing results (arising from reporting biases) for each synthesis assessed. | √  Page 21 and 22 |
| Certainty of evidence | 22 | Present assessments of certainty (or confidence) in the body of evidence for each outcome assessed. | √  Page 20 |
| **DISCUSSION** | | |  |
| Discussion | 23a | Provide a general interpretation of the results in the context of other evidence. | √ |
|  | 23b | Discuss any limitations of the evidence included in the review. | √ |
|  | 23c | Discuss any limitations of the review processes used. | √ |
|  | 23d | Discuss implications of the results for practice, policy, and future research. | √ |
| **OTHER INFORMATION** | | |  |
| Registration and protocol | 24a | Provide registration information for the review, including register name and registration number, or state that the review was not registered. | √ |
|  | 24b | Indicate where the review protocol can be accessed, or state that a protocol was not prepared. | √ |
|  | 24c | Describe and explain any amendments to information provided at registration or in the protocol. | √ |
| Support | 25 | Describe sources of financial or non-financial support for the review, and the role of the funders or sponsors in the review. | √ |
| Competing interests | 26 | Declare any competing interests of review authors. | √ |
| Availability of data, code and other materials | 27 | Report which of the following are publicly available and where they can be found: template data collection forms; data extracted from included studies; data used for all analyses; analytic code; any other materials used in the review. | √ |

*From:*  Page MJ, McKenzie JE, Bossuyt PM, Boutron I, Hoffmann TC, Mulrow CD, et al. The PRISMA 2020 statement: an updated guideline for reporting systematic reviews. BMJ 2021;372:n71. doi: 10.1136/bmj.n71
