# Supplementary material for: Effect of probiotic intake on athletic ability in healthy people: a systematic review and Bayesian meta-analysis
Source: Front Nutr. 2026 Jan 30;13:1731627. doi: 10.3389/fnut.2026.1731627 (PMC12903275; doi:10.3389/fnut.2026.1731627)
Supplement: Supplementary file 1 [file Data_Sheet_1.zip › Supplementary File S0 Search strategy.pdf]

Supplementary File S0 : Search Strategy

PubMed
((Probiotic*) AND (Post-exercise recovery* OR Athletic performance* OR Sports performance* OR Muscle soreness* OR Resistance training* OR Endurance performance* OR Aerobic ability* OR Anaerobic ability* OR Muscle strength* OR Maximum strength* OR Lower body strength* OR Post-exercise fatigue* OR Fatigue*) AND (randomizedcontrolledtrial[Filter])) AND ((((randomized controlled trial*) OR (RCT)) OR (randomized*)))

EBSCO
Probiotic* AND (Post-exercise recovery* or Athletic performance* or Sports performance* or Muscle soreness* or Resistance training* or Endurance performance* or Aerobic ability* or Anaerobic ability* or Muscle strength* or Maximum strength* or Lower body strength* or Post-exercise fatigue* or Fatigue*) AND Probiotic*

Embase
probiotic*:ti,ab,kw AND ('post-exercise recovery*':ti,ab,kw OR 'athletic performance*':ti,ab,kw OR 'sports performance*':ti,ab,kw OR 'muscle soreness*':ti,ab,kw OR 'resistance training*':ti,ab,kw OR 'endurance performance*':ti,ab,kw OR 'aerobic ability*':ti,ab,kw OR 'anaerobic ability*':ti,ab,kw OR 'muscle strength*':ti,ab,kw OR 'maximum strength*':ti,ab,kw OR 'lower body strength*':ti,ab,kw OR 'post-exercise fatigue*':ti,ab,kw OR fatigue*:ti,ab,kw) AND ('randomized controlled trial'/exp OR 'randomized controlled trial':ti,ab OR rct:ti,ab OR randomized*:ti,ab)

Web of Science
1.Probiotic* (Topic) and Preprint Citation Index (Exclude – Database)
2.Post-exercise recovery* (Topic) or Athletic performance* (Topic) or Sports performance* (Topic) or Muscle soreness* (Topic) or Resistance training* (Topic) or Enduranceperformance* (Topic) or Aerobic ability* (Topic) or Anaerobic ability* (Topic) or Muscle strength* (Topic) or Maximum* (Topic) or Strength* (Topic) or Lower body strength* (Topic) or Post-exercise fatigue* (Topic) or Fatigue* (Topic) and Preprint Citation Index (Exclude – Database)
3.randomized controlled trial* (Topic) or RCT (Topic) or randomized* (Topic) and Preprint Citation Index (Exclude – Database)
4.1AND2AND3

Scopus
1.TITLE-ABS-KEY (Probiotic)
2.(TITLE-ABS-KEY (Post-exercise recovery) OR TITLE-ABS-KEY (Athletic performance) O R TITLE-ABS-KEY (Sports performance) OR TITLE-ABS-KEY (Muscle soreness) OR TITL E-ABS-KEY (Resistance training) OR TITLE-ABS-KEY (Enduranceperformance) OR TITLE-ABS-KEY (Aerobic ability) OR TITLE-ABS-KEY (Anaerobic ability) OR TITLE-ABS-KEY (Muscle strength) OR TITLE-ABS-KEY (Maximum) OR TITLE-ABS-KEY (Strength) OR TI TLE-ABS-KEY (Lower body strength) OR TITLE-ABS-KEY (Post-exercise fatigue) OR TITL

E-ABS-KEY (Fatigue))
3.TITLE-ABS-KEY (Probiotic)
4.1AND2NAD3

OVID

1 ("Post-exercise recovery" or "Athletic performance" or "Sports performance" or "Muscle soreness" or "Resistance training" or "Endurance performance" or "Aerobic ability" or "Anaerobic ability" or "Muscle strength" or "Maximum" or "Strength" or "Lower body strength" or "Post-exercise fatigue" or "Fatigue").af.
2 ("randomized controlled trial*" or "RCT" or "randomized*").af.
3 "Probiotic*".af.
4 1 and 2 and 3

(("Post-exercise recovery" or "Athletic performance" or "Sports performance" or "Muscle soreness" or "Resistance training" or "Endurance performance" or "Aerobic capacity" or "Anaerobic capacity" or "Muscle strength" or "Maximum strength" or "Lower body strength" or "Post-exercise fatigue" or "Fatigue") and ("randomized controlled trial*" or "RCT*" or "randomized*") and probiotic*).af.
