# Supplementary material for: Effect of probiotic intake on athletic ability in healthy people: a systematic review and Bayesian meta-analysis
Source: Front Nutr. 2026 Jan 30;13:1731627. doi: 10.3389/fnut.2026.1731627 (PMC12903275; doi:10.3389/fnut.2026.1731627)
Supplement: Supplementary file 1 [file Data_Sheet_1.zip › Supplementary file S3 Risk of Bias in Individual Studies.pdf]

Table 1: <i> Supplement File. Risk of Bias in Individual Studies </i>

Author	Sequence generation	Allocation concealment	Blinding participants	Blinding personnel	Blinding outcome assessment	Incomplete outcome	Selective reporting	Other bias	Overall bias
Afrooz	Low	Low	Low	Low	Low	Low	Low	Low	Low
Fu	Unclear	Unclear	Low	Low	Unclear	Low	Unclear	Low	Unclear
Gutierrez	Low	Low	Low	Low	Unclear	Low	Low	Unclear	Low
HuangA	Unclear	Unclear	Low	Low	Unclear	Low	Unclear	Unclear	Unclear
HuangB	Unclear	Unclear	Low	Low	Unclear	Low	Unclear	Unclear	Unclear
Hudson	Low	Low	Low	Low	Low	Low	Low	Unclear	Low
Imanian	Low	Low	Low	Low	Low	Low	Low	Low	Low
Kurach	Low	Unclear	Low	Low	Low	Low	Low	Unclear	Low
LeeA	Low	Low	Low	Low	Low	Low	Low	Unclear	Low
LeeB	Low	Low	Low	Low	Low	Low	Low	Unclear	Low
LeeCa	Low	Low	Low	Low	Low	Low	Low	Unclear	Low
LeeCb	Low	Low	Low	Low	Low	Low	Low	Unclear	Low
Li	Low	Unclear	Low	Unclear	Unclear	Low	Low	Unclear	Unclear
McDermott	Low	Low	Low	Low	Low	Low	Low	Low	Low
Mohammadi	Low	Low	Low	Low	Low	Low	Low	Unclear	Low
Sadeghi	Unclear	Unclear	Low	Low	Low	Low	Low	Unclear	Low
Salarkia	Low	Unclear	Low	Unclear	Unclear	Low	Low	Unclear	Unclear
Salleh	Unclear	Unclear	Low	Low	Unclear	Low	Unclear	Low	Unclear
Shing	Low	Low	Low	Low	Low	Low	Low	Low	Low
Toohey	Low	Low	Low	Low	Low	Low	Low	Unclear	Low
Townsend	Low	Low	Low	Low	Low	Low	Low	Low	Low
Zarzecka	High	High	Low	Low	Low	Unclear	Unclear	Unclear	High
