# Supplementary material for: Effect of probiotic intake on athletic ability in healthy people: a systematic review and Bayesian meta-analysis
Source: Front Nutr. 2026 Jan 30;13:1731627. doi: 10.3389/fnut.2026.1731627 (PMC12903275; doi:10.3389/fnut.2026.1731627)
Supplement: Supplementary file 1 [file Data_Sheet_1.zip › Supplementary file S5 Forest plots.docx]

**Forest diagram of probiotic sub models**

**
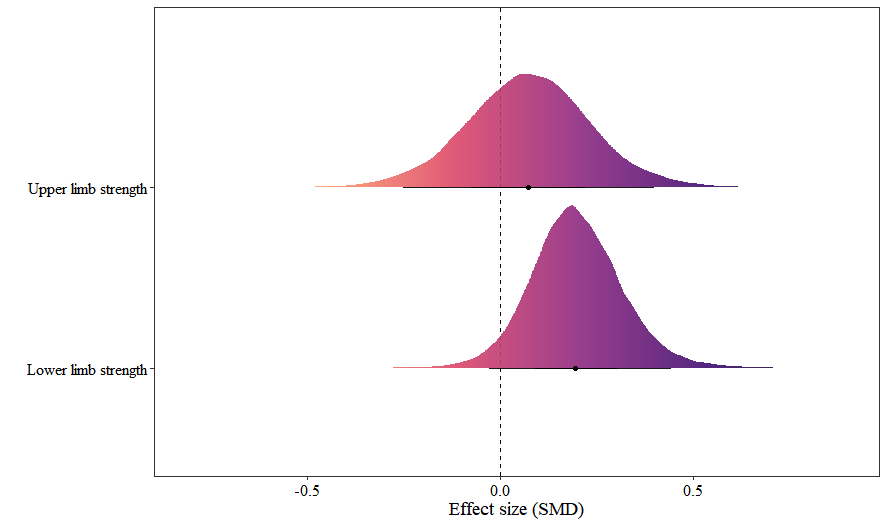
**

**Figure 1.** The Forest Plot in Muscle strength model


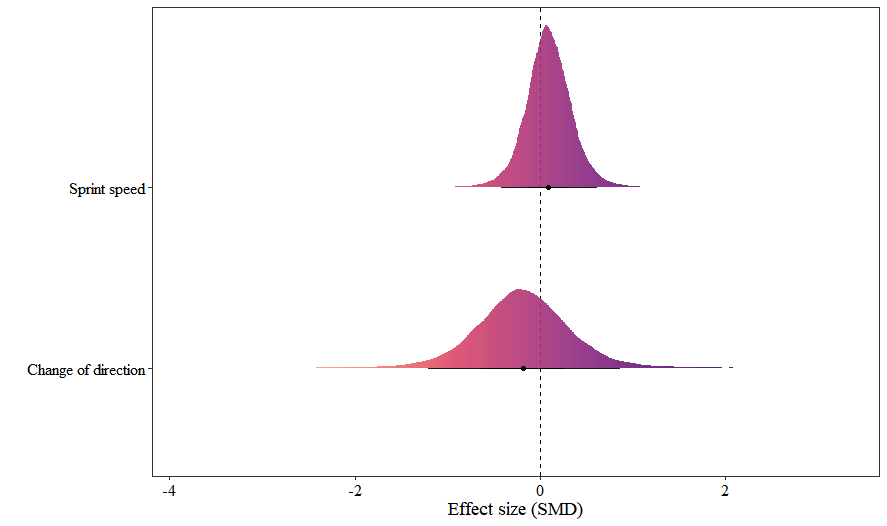


**Figure 2.** The Forest Plot in Agility model


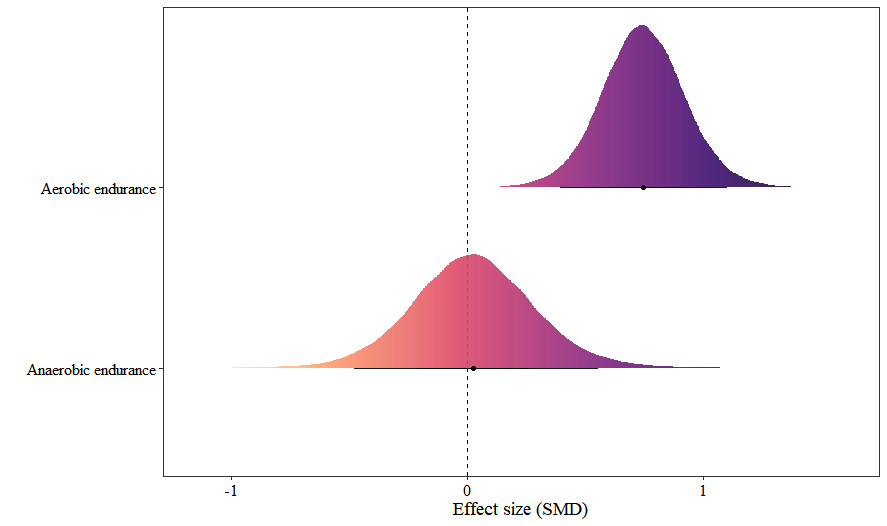


**Figure 3.** The Forest Plot in Endurance model


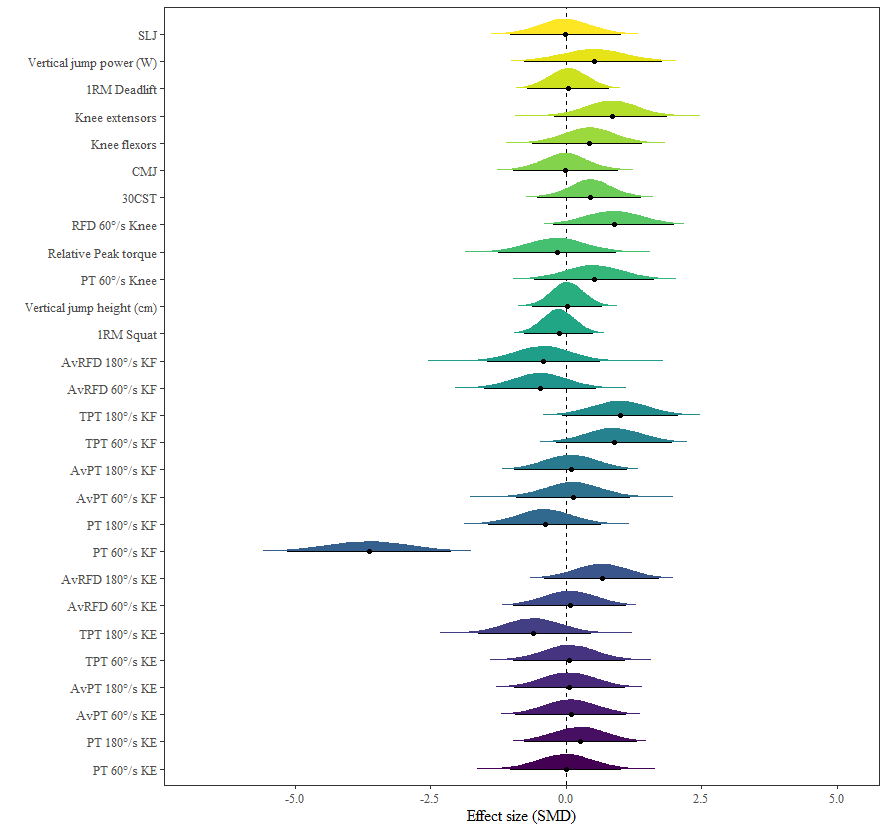


**Figure 4.** The Forest Plot in Lower limb strength model


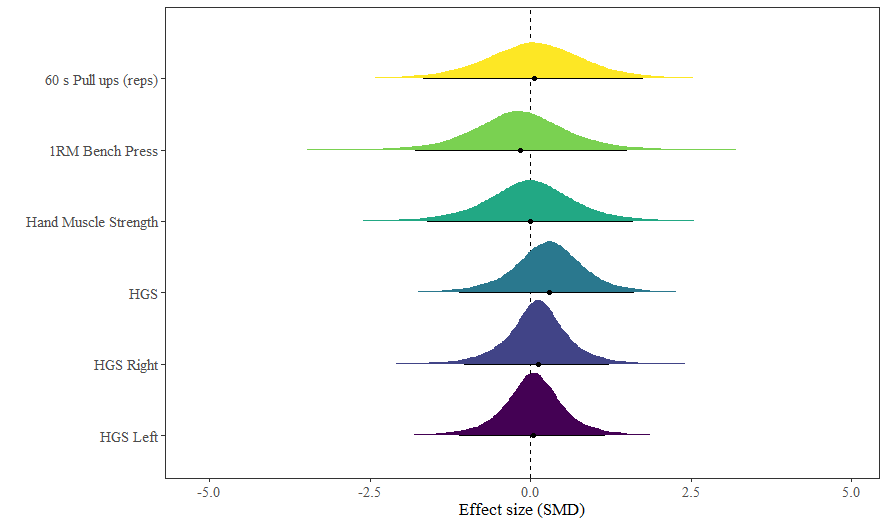


**Figure 5.** The Forest Plot in Upper-limb strength model

**
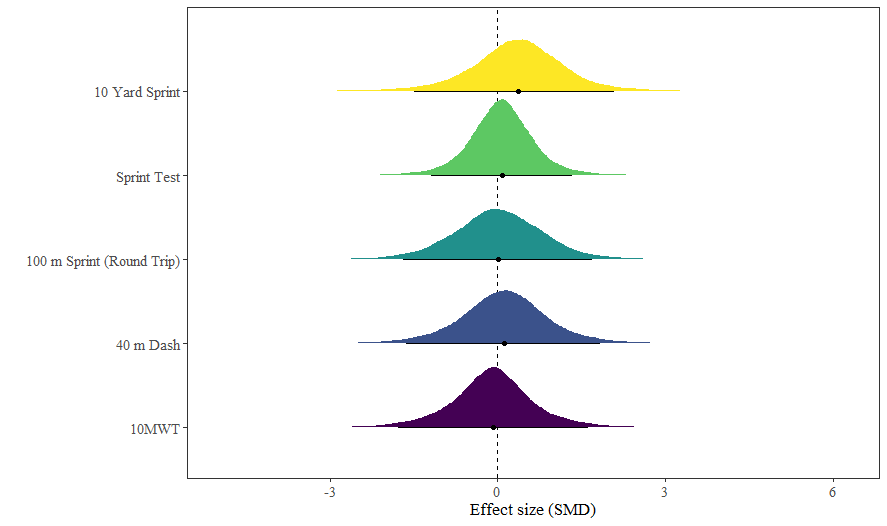
**

**Figure 6.** The Forest Plot in Sprint speed model


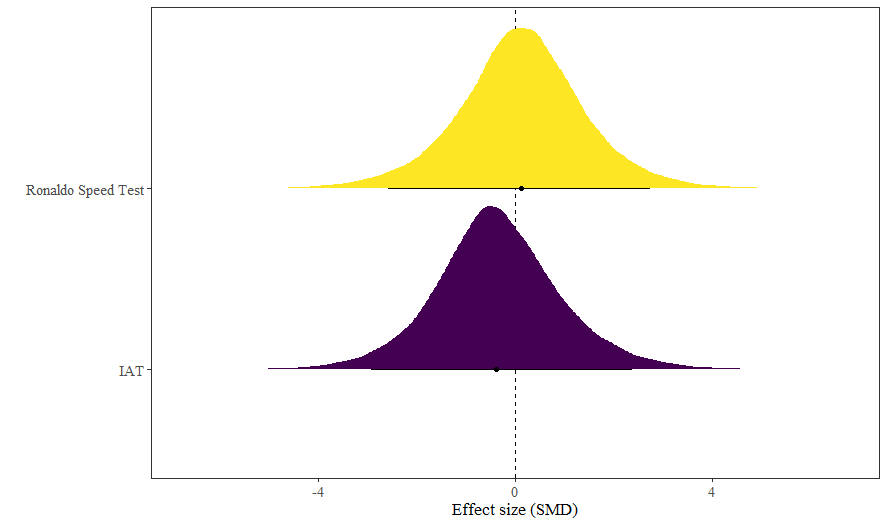


**Figure 7.** The Forest Plot in Change of direction model


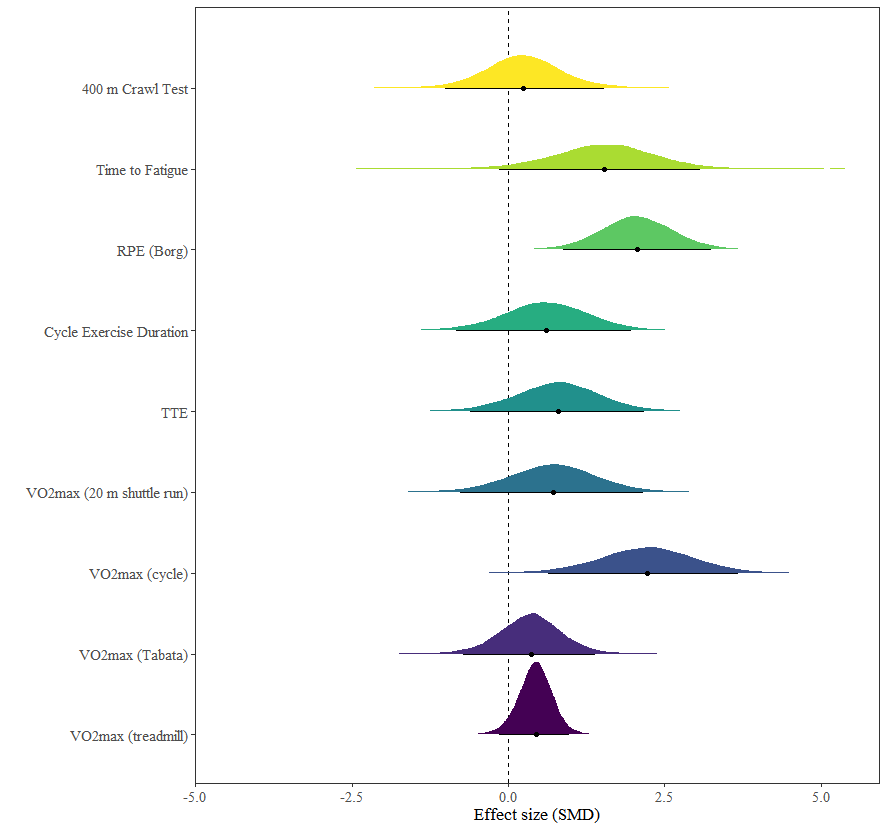


**Figure 8.** The Forest Plot in Aerobic endurance model.


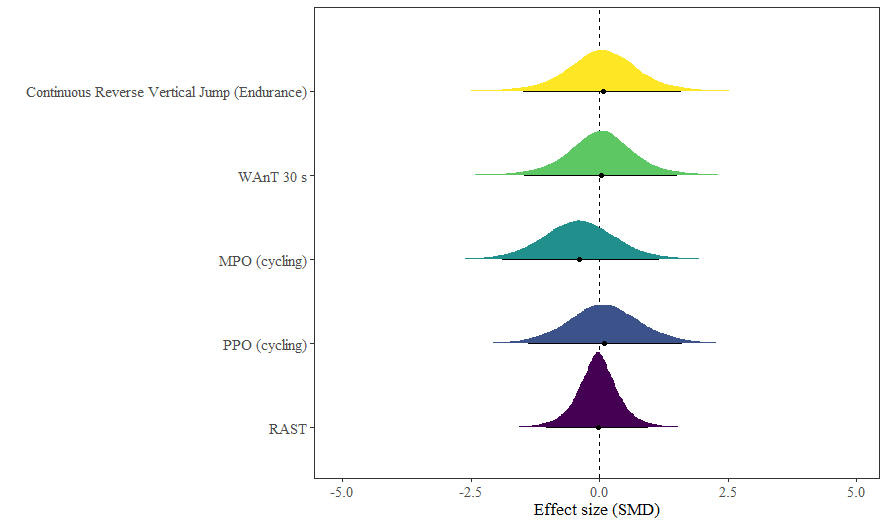


**Figure 9.** The Forest Plot in Anaerobic endurance model


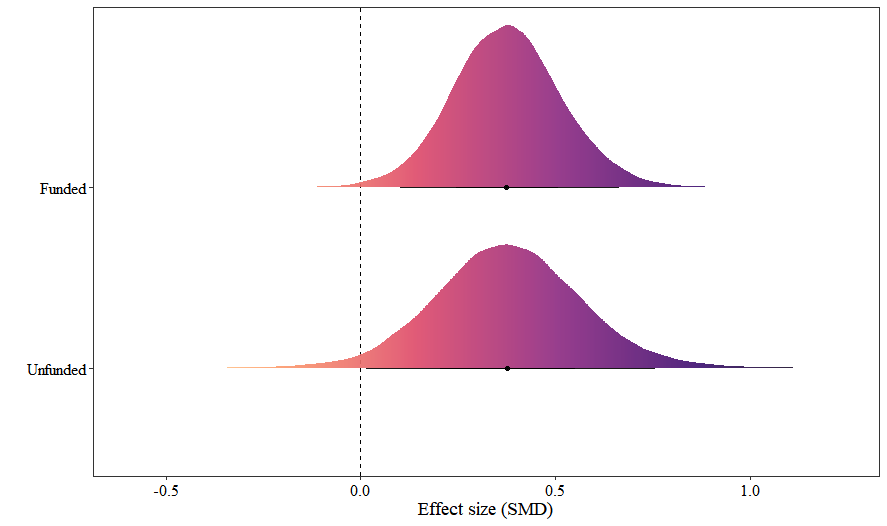


**Figure 10.** The Forest Plot in Financial support model


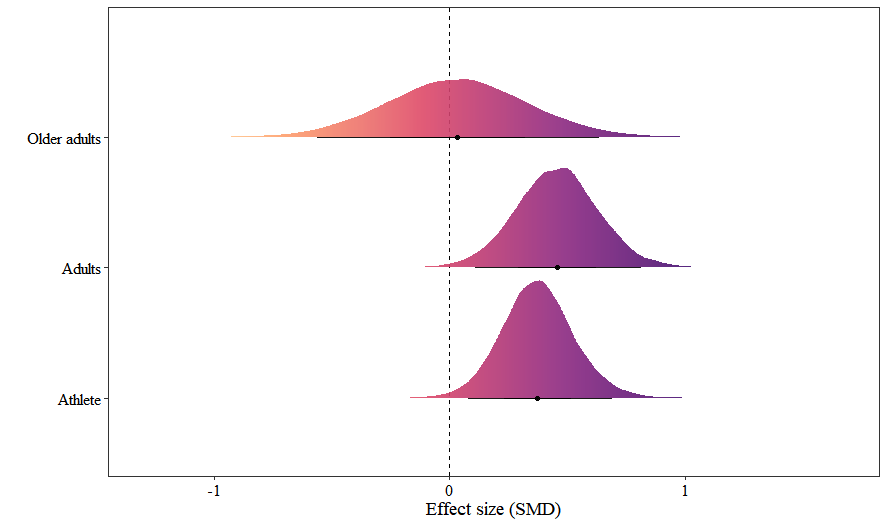


**Figure 11.** The Forest Plot in Demographic model


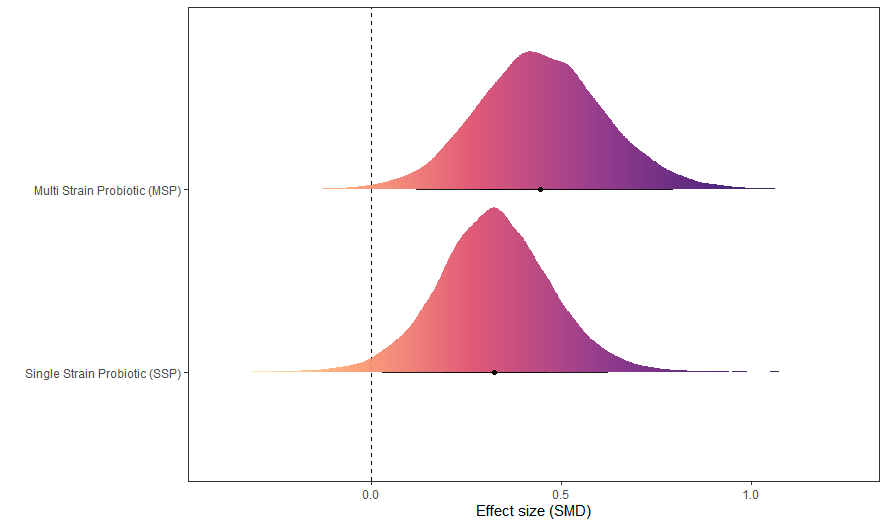


**Figure 12.** The Forest Plot in Probiotic formulation type model


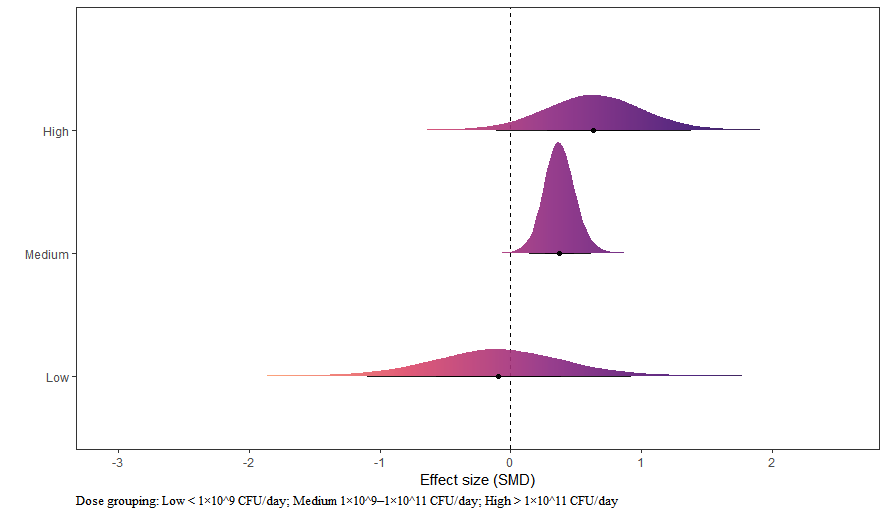


**Figure 13.** The Forest Plot in Probiotic dosage model


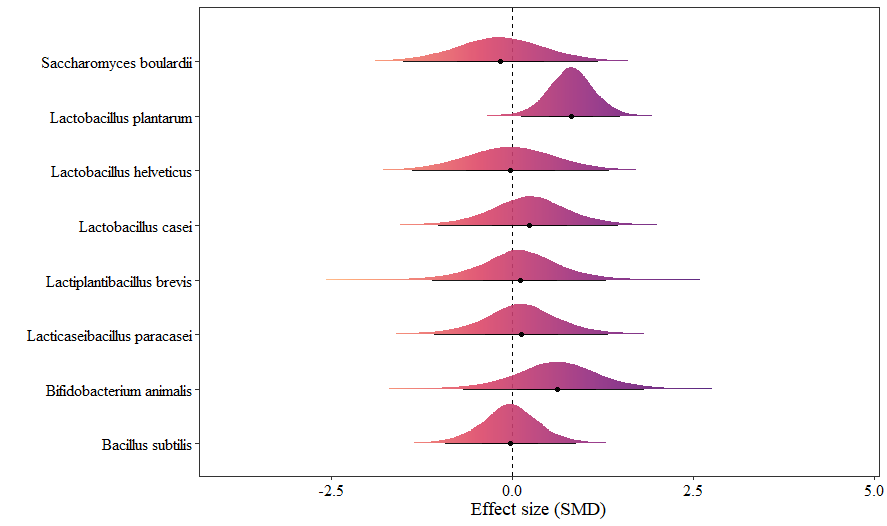


**Figure 14.** The Forest Plot in Single strain model
